# PsRGL1 negatively regulates chilling- and gibberellin-induced dormancy release by PsF-box1-mediated targeting for proteolytic degradation in tree peony

**DOI:** 10.1093/hr/uhad044

**Published:** 2023-03-13

**Authors:** Linqiang Gao, Demei Niu, Tianyu Chi, Yanchao Yuan, Chunying Liu, Shupeng Gai, Yuxi Zhang

**Affiliations:** College of Life Sciences, Qingdao Agricultural University, Qingdao 266109, China; University Key Laboratory of Plant Biotechnology in Shandong Province, Qingdao 266109, China; College of Life Sciences, Qingdao Agricultural University, Qingdao 266109, China; University Key Laboratory of Plant Biotechnology in Shandong Province, Qingdao 266109, China; College of Life Sciences, Qingdao Agricultural University, Qingdao 266109, China; University Key Laboratory of Plant Biotechnology in Shandong Province, Qingdao 266109, China; College of Life Sciences, Qingdao Agricultural University, Qingdao 266109, China; University Key Laboratory of Plant Biotechnology in Shandong Province, Qingdao 266109, China; College of Life Sciences, Qingdao Agricultural University, Qingdao 266109, China; University Key Laboratory of Plant Biotechnology in Shandong Province, Qingdao 266109, China; College of Life Sciences, Qingdao Agricultural University, Qingdao 266109, China; University Key Laboratory of Plant Biotechnology in Shandong Province, Qingdao 266109, China; College of Life Sciences, Qingdao Agricultural University, Qingdao 266109, China; University Key Laboratory of Plant Biotechnology in Shandong Province, Qingdao 266109, China

## Abstract

Tree peony bud endodormancy is a common survival strategy similar to many perennial woody plants in winter, and the activation of the GA signaling pathway is the key to breaking endodormancy. GA signal transduction is involved in many physiological processes. Although the GA-GID1-DELLA regulatory module is conserved in many plants, it has a set of specific components that add complexity to the GA response mechanism. DELLA proteins are key switches in GA signaling. Therefore, there is an urgent need to identify the key DELLA proteins involved in tree peony bud dormancy release. In this study, the prolonged chilling increased the content of endogenously active gibberellins. PsRGL1 among three DELLA proteins was significantly downregulated during chilling- and exogenous GA_3_-induced bud dormancy release by cell-free degradation assay, and a high level of polyubiquitination was detected. Silencing *PsRGL1* accelerated bud dormancy release by increasing the expression of the genes associated with dormancy release, including *PsCYCD, PsEBB1*, *PsEBB3*, *PsBG6*, and *PsBG9*. Three F-box protein family members responded to chilling and GA_3_ treatments, resulting in *PsF-box1* induction. Yeast two-hybrid and BiFC assays indicated that only PsF-box1 could bind to PsRGL1, and the binding site was in the C-terminal domain. *PsF-box1* overexpression promoted dormancy release and upregulated the expression of the dormancy-related genes. In addition, yeast two-hybrid and pull-down assays showed that PsF-box1 also interacted with PsSKP1 to form an E3 ubiquitin ligase. These findings enriched the molecular mechanism of the GA signaling pathway during dormancy release, and enhanced the understanding of tree peony bud endodormancy.

## Introduction

Bud dormancy is a survival strategy for perennial woody plants in adverse environments in winter, and tree peony bud dormancy is known as endodormancy. Forcing culture in winter has become an important component of the tree peony industry, and artificial chilling and exogenous gibberellin treatments are common measures to break dormancy with a high efficiency during production. In forcing culture practice, tree peony often fails to flower or flowers poorly because of an incomplete understanding of dormancy release mechanism in practice. Therefore, there is an increasing urgency for further studies on the mechanism of tree peony bud dormancy release.

Advances regarding the physiological and molecular mechanism of woody plant bud dormancy release have recently resulted in important breakthroughs [1, 2]. Key flowering regulators, such as CONSTANS (CO), FLOWERING LOCUS T (FT), and PHYTOCHROMES (PHYs), are involved not only in flowering time regulation but also in short-day (SD)-induced growth cessation [3–5]. *Dormancy-associated MADS-box 6* (*DAM6*) inhibits the bud break at dormancy and budbreak stages in apple [6]. In poplar, the overexpression of CENTRORADIALIS (CEN)/TERMINAL FLOWER 1 (TFL1) delays bud dormancy release [7], while EARLY BUD BREAK 1 (EBB1), EBB3, and APETALA2/ethylene-like responsive factors positively promote bud break. EBB3 can positively and directly regulate *CYCD3*, which is a regulator of the G1/S phase transition that promotes cell proliferation. The induction and maintenance of dormancy are accompanied by the blockage of plasmodesmata (PD) with callose, while dormancy release and regrowth require reopening of the transport channels, and β-1,3-D-glucanase is the crucial component fulfilling this function [8]. In tree peony, *PsBG6* responds to chilling and *PsBG9* responds to exogenous GA_3_ treatment to hydrolyze callose [9].

Several studies have found that bud dormancy release and budbreak depend on sufficient gibberellin (GA) threshold levels [10, 11]. Differentially expressed genes (DEGs) associated with chilling- and GA-induced dormancy release, as well as microRNAs and proteins [13, 14], are enriched in the GA synthesis and signaling pathways [10–13], and the activation of the GA signaling pathway is the key to breaking bud dormancy in tree peony [12]. The key genes associated with gibberellin synthesis, including *GA 20-oxidase* gene (*GA20ox*) and *GA 3-oxidase* gene (*GA3ox*), are induced, and a *GA-stimulated transcripts gene 1* (*GAST1*) is upregulated [15], accompanied by downregulation of the *GA2ox*, during the transition from dormancy to dormancy release [10, 16].

The central GA signaling components in plants have been identified, including the GA receptor GIBBERELLIN-INSENSITIVE DWARF1 (GID1), DELLA, GID2 or Sleepy1 (SLY1), which display not only overlapping but also some distinct functions in GA responses [17, 18]. In *Arabidopsis*, there are three GID1s, five DELLAs (RGA, GAI, RGA-LIKE1 (RGL1), RGL2, and RGL3), and two SLY-like proteins (SLY1 and SNE). Two GID2-like proteins (GID2 and SNE), one GID1 protein and one DELLA protein have been identified in rice [19]. In grape, there are two GID1s, three DELLAs, and three SLY-like proteins [20].

It is well known that active GA binds to GID1, which then bind to DELLA to form a GA-GID1-DELLA complex. DELLA is then recruited to the SCF^SLY1/GID2^ E3 ubiquitin ligase for polyubiquitination and subsequent degradation by the 26S proteasome, resulting in plants exhibiting GA responses [21, 22]. In peach, DELLA2 is the key member that transmits gibberellin signaling, thereby modulating the endodormancy release of leaf buds [23]. Among the three DELLAs in Japanese apricot, PmRGL2 plays a negative role in bud dormancy release [24]. Both *GID2* and *SLY1* encode putative F-box proteins with a conserved F-box domain, and this domain interacts with SKP1 proteins to form an E3 ubiquitin ligase with cullin and RBX1 proteins [25], wherein the C-terminal regulatory domain is responsible for binding DELLA proteins [26]. Although the GA-GID1-DELLA regulatory module is conserved in many plants, each has a set of specific GA signaling components, which adds complexity to the GA response mechanism. In tree peony, the components of GA signaling involved in bud dormancy release are unknown, and there is urgent need to identify the key DELLA protein involved during bud dormancy release, which will provide insights into the molecular mechanism of GA signaling during bud dormancy release in tree peony.

## Results

### PsRGL1 negatively regulates bud dormancy release by repressing GA signaling

Recent studies indicated that effective chilling accumulation is necessary to break tree peony bud dormancy, and 21 d of chilling is sufficient for dormancy release of ‘Luhehong’ [27]. Moreover, exogenous GA treatment was found to significantly promote dormancy release and bud burst, as well as significantly increase the content of endogenous active GAs [10], suggesting that GA signaling is involved in tree peony bud dormancy release. In this study, the contents of endogenously active gibberellins during chilling-induced dormancy release were detected by GC–MS, and the contents of endogenously active GA_1_ and GA_4_ were found to be increased with prolonged chilling duration (Fig. S1, see online supplementary material), indicating that prolonged chilling activates the GA pathway during tree peony bud dormancy release.

DELLA proteins are known to repress the GA signaling pathway. A total of five DELLA proteins of *Arabidopsis* (AtGAI, AtRGA, AtRGL1, AtRGL2, and AtRGL3) were used as seed sequences, and *DELLA*-like genes in *Paeonia suffruticosa* genome were surveyed by local BLAST. A total of three *DELLA*-like genes were obtained after redundancy removal, among which *PsDELLA1* had a 1620 bp ORF encoding 540 aa (GenBank accession no. OP272869), *PsDELLA2* had a 1863 bp ORF encoding 621 aa (GenBank accession no. OP272870), and *PsDELLA3* had a 1848 bp ORF encoding 616 aa (GenBank accession no. OQ224301) (File SS1, see online supplementary material). They had the conserved DELLA and GRAS domains and were located at aa 34–100 and 161–524 in PsDELLA1, aa 45–112 and 249–610 in PsDELLA2, and aa 54–120 and 247–605 in PsDELLA3 (Fig. S2, see online supplementary material). The phylogenetic tree showed that the putative DELLA proteins were clustered into three sub-clades of DELLA proteins, which were designated as PsRGL1 (44.33% with AtRGL1), PsGAI1 (25.51% identity with PmGAI), and PsGAIP-B (42.54% identity with PtGAIP-B) (Fig. S3, see online supplementary material).

**Figure 1:**
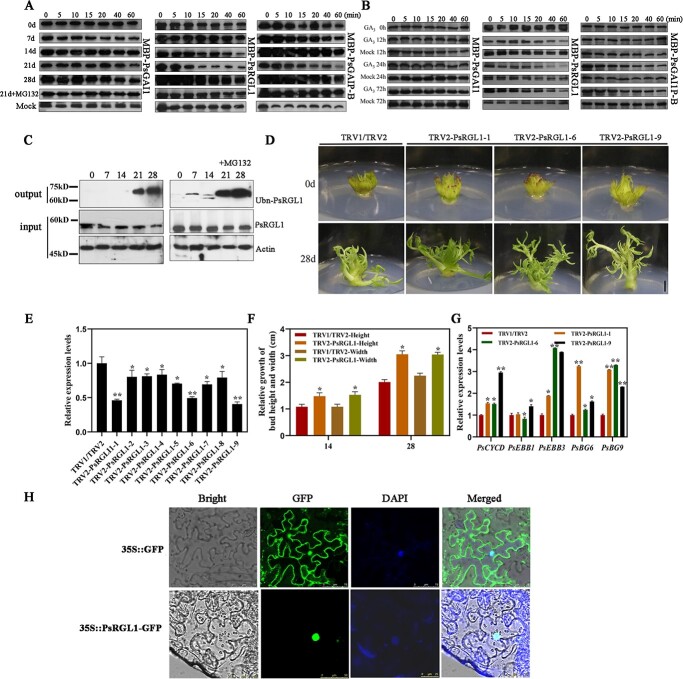
Identification of PsDELLA proteins associated with dormancy release in tree peony. **A** Cell-free degradation assays of three PsDELLAs protein after chilling. The tree peony buds after different chilling-treated days were used to extract total proteins, which were incubated with recombinant MBP-PsRGL1, MBP-PsGAI1, and MBP-PsGAIP-B. 21 d + MG132 (10 μM) represents the total protein of 21 chilling buds incubated with MBP-PsRGL1, MBP-PsGAI1, and MBP-PsGAIP-B containing MG132 (10 μM). Mock represented only recombinant MBP-PsRGL1 and MBP-PsGAI1 without total tree peony protein under incubation conditions, respectively. **B** Cell-free degradation assays of three PsDELLAs protein after exogenous GA_3_ treatment. **C** Polyubiquitination of PsRGL1 during chilling-induced bud dormancy release. The total protein from buds after different chilling days was probed with anti-PsRGL1 antibody, and total ubiquitinated PsRGL1 was detected with anti-Ub antibody in the output. The accumulations of PsRGL1 and actin were tested in the input. **D** Morphological changes of TRV2-*PsRGL1* transgenic buds after being transformed for 28 d. Scale bar, 1.0 cm. **E** Relative expression level of *PsRGL1* in *PsRGL1-*silenced buds by qRT-PCR after being infected for 7 d. **F** Relative expression levels of genes, including *PsCYCD, PsEBB1*, *PsEBB3*, *PsBG6*, and *PsBG9*, associated with tree peony dormancy release by qRT-PCR in *PsRGL1-*silenced buds after being transformed for 7 d. **G** Relative growth in terms of height and width in in *PsRGL1-*silenced buds after being transformed for 14 d and 28 d. Data are represented as the means ± standard deviation (SD) of six replicates. ^*^P < 0.05; ^**^P < 0.01. **H** Subcellular localization of PsRGL1 by fluorescence microscope at an excitation wavelength of 488 nm.

qRT-PCR was used to detect the expression patterns of these three *PsDELLA* genes, and the results showed that they responded to chilling and exogenous gibberellin treatments, and these results were fundamentally consistent with those of the microarray (Fig. S4, see online supplementary material). Unfortunately, we could not identify the key *PsDELLA* member that regulates dormancy release at the transcriptional level. Therefore, a cell-free degradation assay was used to identify the DELLA member that tended to be downregulated during chilling-induced bud dormancy release. Total proteins were extracted from chilling-treated tree peony buds and incubated with recombinant MBP-PsRGL1, MBP-PsRGAI1, and MBP-PsGAIP-B. In terms of the controls, MBP-PsRGL1, MBP-PsGAI1, and MBP-PsGAIP-B remained stable for prolonged incubation times when no tree peony protein was added. PsRGL1 was downregulated in 14 d chilling-treated buds after incubation for 60 min, and its downregulation was more significant in 21 d chilling-treated buds after incubation for 10 min. PsGAIP-B showed slight downregulation in buds after chilling for 21 d, PsGAI1 did not exhibit significant changes during chilling-induced bud dormancy release. After the addition of MG132, no significant changes were observed (Fig. 1A). After exogenous GA_3_ treatment, PsRGL1 was significantly downregulated from 12 h until 24 h, although the amount of PsGAI1 and PsGAIP-B proteins remained stable (Fig. 1B). In addition, the PsRGL1 protein levels during chilling-treatment dormancy release were significantly downregulated after 14 d to 21d of chilling (Fig. 1A). Based on our recent study, 21 d of chilling is adequate for ‘Luhehong’ to break dormancy, and the state of buds after chilling for 14 d is a transition stage from endodormancy to endodormancy release [27]. Therefore, PsRGL1 was used for further study. The PsRGL1 protein level decreased during dormancy release, suggesting a decline caused by polyubiquitination. Therefore, the polyubiquitination signal was detected during chilling-induced bud dormancy release, and a high level of PsRGL1 polyubiquitination was observed after 21 d and 28 d of chilling treatment. After the addition of MG132, higher levels of polyubiquitination were detected, consistent with the inhibition of the 26S proteasome by MG132 (Fig. 1C). Taken together, these results indicated that PsRGL1 was a reliable DELLA member that functions as a repressor of the GA pathway during bud dormancy release in tree peony.

To identify the function of PsRGL1 during chilling-induced bud dormancy release, the specific fragment of *PsRGL1* was used to construct the TRV2-PsRGL1 silencing vector based on the alignment of PsRGL1 and other DELLA proteins (Fig. S2, see online supplementary material), which was transformed into *Agrobacterium* EHA105, and then used to infect tree peony flower buds after 7 d of chilling. The expression level of *PsRGL1* was analysed by qRT-PCR after normal culture for 7 d, and it was significantly downregulated in nine *PsRGL1*-silenced buds (Fig. 1E). From 14 d to 28 d, the relative growth rate was measured, and TRV2-PsRGL1 buds grew taller and wider (Fig. 1D and F). TRV2-PsRGL1–1, −6, and − 9 buds with a silencing efficiency of more than 50% were used to analyse the genes associated with dormant release, including *PsCYCD*, *PsEBB1*, *PsEBB3*, *PsBG6*, and *PsBG9.* The expression of these genes was found to be significantly increased, with the expression level of *PsEBB3* increasing more than four-fold in these three TRV2-PsRGL1 buds (Fig. 1G). Therefore, *PsRGL1* negatively regulated bud dormancy release.

**Figure 2:**
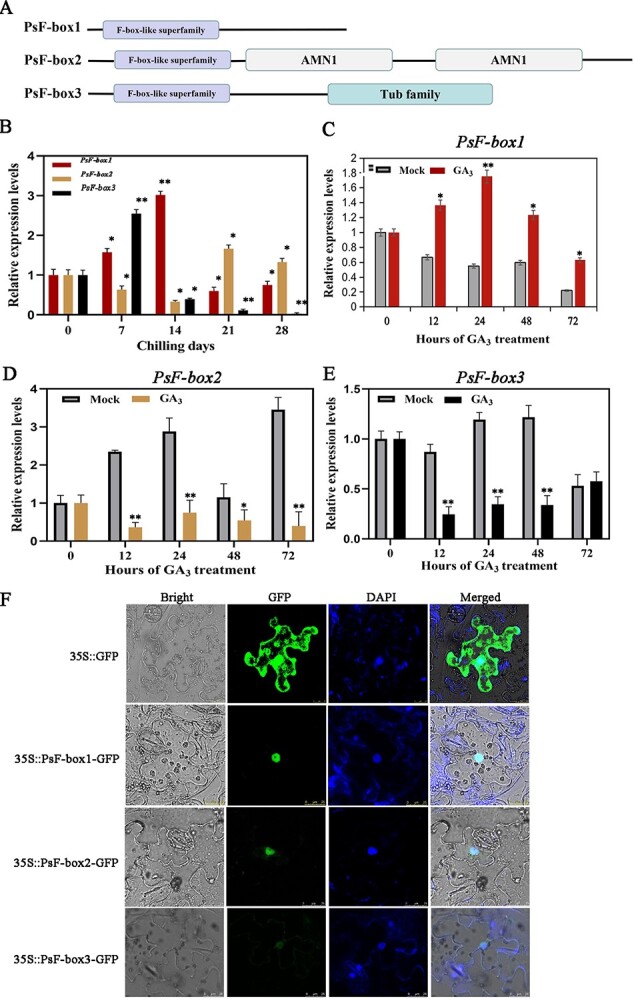
Domain arrangement of the putative PsF-box proteins and the expression patterns of three *PsF-box* genes during chilling- and GA_3_-induced dormancy release. **A** Linear representation of the domain arrangement of the putative PsF-box proteins, of which PsF-box1 had a 567 bp ORF encoding 189 aa, PsF-box2 had a 1617 bp ORF encoding 539 aa, and PsF-box3 had a 777 bp ORF encoding 259 aa. Boxes with different colors represent different domains. **B** Expression patterns of *PsF-box1*, *PsF-box2*, and *PsF-box3* after different chilling durations. **C**–**E** Expression of *PsF-box1*, *PsF-box2*, and *PsF-box3* after exogenous GA_3_ treatment, respectively. **F** Subcellular localization of PsF-box1, PsF-box2, and PsF-box3 by fluorescence microscope with an excitation wavelength of 488 nm. Data represent the mean ± SD of six replicates. ^*^P < 0.05; ^**^P < 0.01.

Dill *et al.* found that GID2 protein targets DELLA proteins in the nucleus [28]. Therefore, the subcellular localization of the putative PsRGL1 protein was predicted using ProtComp software, the results of which indicated that PsRGL1 may be located in the nucleus. To determine the subcellular location of PsRGL1, the fusion expression vector *PsRGL1-GFP* was constructed and instantaneously transformed into tobacco leaves, with *35S::GFP* as a control. Laser scanning confocal microscopy showed that PsRGL1 was located in the nucleus and overlapped with the DAPI fluorescence (Fig. 1H).

**Figure 3:**
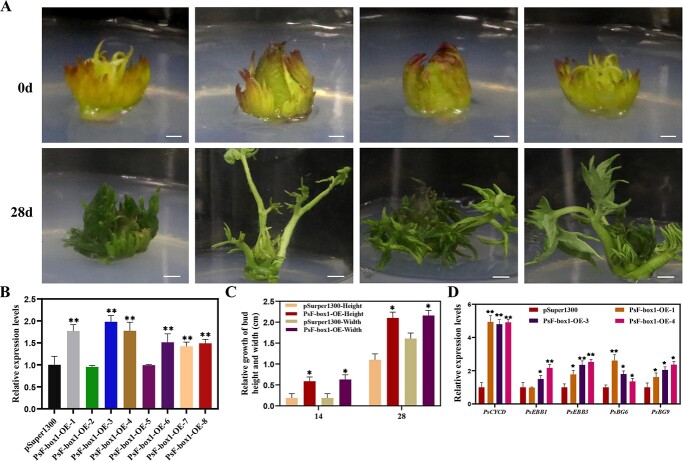
Morphological changes and expression levels of genes associated with bud dormancy release in tree peony after overexpression of *PsF-box1*. **A** Morphological changes of overexpressing *PsF-box1* buds after being transformed for 28 d. Scale bar, 1.0 cm. **B** Relative expression level of *PsF-box1* by qRT-PCR after being infected for 7 d. **C** Relative growth in terms of the height and width of *PsF-box1*-OE buds after being transformed for 14 d and 28 d. The data are presented as means ± the SD of six replicates for the relative expression level. **D** Relative expression levels of genes including *PsCYCD*, *PsEBB1*, *PsEBB3*, *PsBG6*, and *PsBG9*, associated with tree peony dormancy release by qRT-PCR in *PsF-box1*-OE buds after being transformed for 7 d. Data represent the mean ± SD of six replicates. ^*^P < 0.05; ^**^P < 0.01.

### Cloning and expression analysis of *PsF-boxes*

A total of 96 F-box family members were obtained from *P. suffruticosa* genome. Among them, there were 16 members responding to chilling, and 23 members responded to GA_3_ treatment, only three F-box members responded not only to chilling but also to GA_3_ treatment [10, 12]. *PsF-box1* was comprised of 834 bp containing an 8 bp 5’ UTR, a 205 bp 3’ UTR, and a 567 bp ORF encoding 189 aa (GenBank accession no. JI545895); *PsF-box2* was comprised of 2067 bp with a 216 bp 5’ UTR, a 234 bp 3’ UTR and a 1617 bp ORF encoding 539 aa (GenBank accession no. JI447773); *PsF-box3* was comprised of 1011 bp with a 234 bp 5’ UTR, and a 777 bp ORF encoding 259 aa (GenBank accession no. JI445105) (File SS1, see online supplementary material). A search of the Pfam protein database revealed that these three F-box proteins contained one conserved F-box domain. In addition, F-box2 had two AMN1 domains, and F-box3 had one Tub family domain (Fig. 2A). The phylogenetic tree showed that PsF-box1 was clustered into the GID2 branch with 75.30% and 72.46% similarity to VvGID2 and CpGID2, respectively, which is consistent with the homology alignment (Fig. S5, see online supplementary material). The other two F-box proteins, F-box2 and F-box3, were clustered into two other branches. After the alignment of tree peony F-box1 proteins with other known F-box proteins, the sequence deviation was mainly located in the N-terminal domain and the middle of the C-terminal domain. After the alignment of the PsF-box1 protein and other GID2 proteins in other plants, three domains were found to have a relatively high sequence similarity, namely F-box, GGF, and LSL domains, which are typical domains associated with the F-box among the subunits of the SCF E3 ubiquitin ligase complex (Fig. S6, see online supplementary material).

qRT-PCR was used to analyse the expression patterns of these genes. During chilling-induced dormancy release, *PsF-box1* was upregulated and reached a peak after 14 d of chilling treatment. *PsF-box2* was significantly downregulated after 7 d of chilling until 14 d, followed by significant upregulation until 28 d. *PsF-box3* was significantly induced after 7 d, followed by pronounced downregulation until 28 d (Fig. 2B). After exogenous GA_3_ treatment, only *PsF-box1* was upregulated after 12 h and peaked after 24 h, whereas the other two genes were inhibited by exogenous GA_3_, which indicated that *PsF-box1* could be induced by exogenous GA_3_ (Fig. 2C–E). Finally, the subcellular localizations of the three putative PsF-box proteins were determined using laser scanning confocal microscopy, which revealed that the GFP fluorescence overlapped with the DAPI staining, indicating that these PsF-box proteins were located in the nucleus (Fig. 2F).

### *PsF-box1* accelerates bud dormancy release

To evaluate the function of *PsF-box1*, which is an important component of the SCF complex during chilling-induced dormancy release, the pSuper1300*-PsF-box1* overexpression vector was constructed and transformed into *Agrobacterium* EHA105, which was used to infect the tree peony buds after 7 d of chilling. After culturing for 7 d, the *PsF-box1* expression levels were analysed by qRT-PCR. In a total of eight randomly selected *PsF-box1* overexpression (OE) buds, the expression levels of *PsF-box1* in six buds were significantly upregulated. *PsF-box1*-OE-1, 3, and 4 were used to analyse the expression of genes associated with dormancy release, including *PsCYCD*, *PsEBB1*, *PsEBB3*, *PsBG6*, and *PsBG9*. The expression levels of these genes were significantly increased, except for *PsEBB1* in *PsF-box1*-OE-1, where the expression level of *PsCYCD* increased almost five-fold in three *PsF-box1*-OE buds (Fig. 3B and D). After culturing for 14 d, the relative growth rate was measured, and the *PsF-box1*-OE buds were higher and wider (Fig. 3A and C). These results indicated that *PsF-box1* promoted bud dormancy release.

### PsRGL1 interacts with PsF-box1 protein

**Figure 4:**
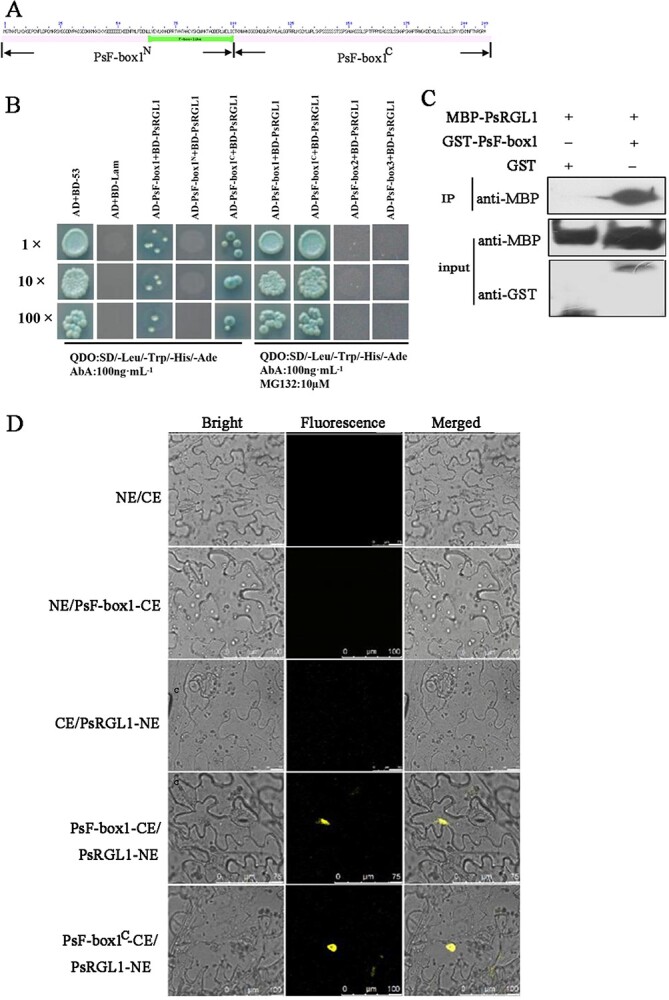
Interaction between PsRGL1 and PsF-box proteins. **A** Outline of PsF-box1 showing the location of the F-box domain. PsF-box1^N^ is the fragment with the F-box domain, PsF-box1^C^ is the fragment without the F-box domain. **B** Yeast two-hybrid assay analysis of PsF-box proteins and PsRGL1 in QDO medium with 100 ng.mL^−1^ AbA and 10 μM MG132. PsF-box1^N^ and PsF-box1^C^ represented the fragments with and without F-box domain, respectively. Scale bar, 100 μm. **C** Interaction between PsF-box1 and PsRGL1 using pull-down assay. **D** BiFC analysis of the interaction between PsF-box1 and PsRGL1 in *N. benthamiana* leaves. PsF-box1 protein was truncated into two fragments, PsF-box1^N^ and PsF-box1^C^, which represent the fragments with and without an F-box domain, respectively. Scale bar, 100 μm.

PsRGL1 was a key DELLA member, exhibiting a downregulation pattern during dormancy release, as well as high levels. However, whether its degradation is mediated by E3 ubiquitin ligase activity is not yet known. *GID2* and *SLY1*, which are important SCF E3 ubiquitin ligase components, are known to be encoded by *F-box* genes. Therefore, a yeast two-hybrid assay was carried out to survey the interactions between PsRGL1 and these three candidate PsF-box proteins. The recombinant plasmids pGBKT7-PsRGL1 and pGADT7-PsF-box1 were co-transformed into yeast Y2HGold competent cells and then cultured on SD/−Leu/−Trp/-His/−Ade with 100 ng.mL^−1^ AbA medium. A weak interaction signal was observed, and no signal was obtained after co-transformation of pGBKT7-PsRGL1 and pGADT7-PsF-box2, pGBKT7-PsRGL1 and pGADT7-PsF-box3. As PsRGL1 may be degraded by the 26S proteasome after being polyubiquitinated, MG132 was added, and the results showed that the yeast grew well on QDO (SD/−Leu/−Trp/-His/−Ade with 100 ng.mL^−1^ AbA) medium after the co-transformation of pGBKT7-PsRGL1 and pGADT7-PsF-box1. However, the transformants of pGBKT7-PsRGL1 and pGADT7-PsF-box2, and pGBKT7-PsRGL1 and pGADT7-PsF-box3 did not grow (Fig. 4B). To identify the specific binding domain, PsF-box1 was truncated to generate two fragments (PsPsF-box1^N^ and PsF-box1^C^) (Fig. 4A). The yeast cells grew after co-transformation with pGADT7-PsRGL1 and pGBKT7-PsF-box1^C^, but not after co-transformation with pGADT7-PsRGL1 and pGBKT7-PsF-box1^N^, further confirming that only PsRGL1 and PsF-box1 interacted, indicating that specific binding domain for this interaction was the C-terminal domain of PsF-box1 (Fig. 4B).

To further confirm the interaction between PsF-box1 and PsRGL1, MBP-PsRGL1 and GST-PsF-box1 infusion proteins were induced and purified. The results of the pull-down assay revealed strong signals after co-incubation with recombinant MBP-PsRGL1 and PsF-box1 proteins (Fig. 4C). In addition, a BiFC assay was performed to verify the interaction, and the YFP fluorescence signal was detected after co-transformation of PsRGL1 with PsF-box1 when MG132 was added. The fluorescence signal was also observed after co-transformation with PsF-box1^C^, but no YFP fluorescence signal was observed in the PsF-box1^N^ group (Fig. 4D).

### PsF-box1 is an SCF complex component that interacts with an AtASK1 homolog

**Figure 5:**
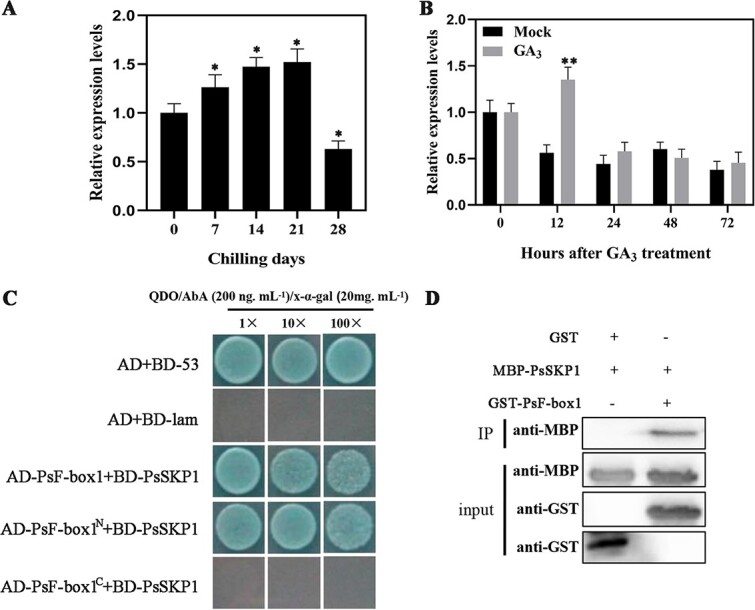
The expression levels of *PsSKP1* during chilling- and GA_3_-induced bud dormancy release, and negative regulation of chilling-induced dormancy release in tree peony buds by interaction between PsF-box1, PsSKP1, and PsRGL1. **A** and **B** Expression of *PsSKP1* after chilling and exogenous GA_3_ treatment by qRT-PCR. **C** Interaction between PsF-box1 and PsSKP1 by yeast two-hybrid assay in QDO (SD/−Leu/−Trp/-His/−Ade) medium with AbA of 200 ng$ \cdot$ mL^−1^ and X-α-gal of 20 mg.mL^−1^. **D** Interaction between PsF-box1 and PsSKP1 by pull-down assay. Data represent the mean ± SD of six replicates. ^*^P < 0.05; ^**^P < 0.01.

The response of *PsF-box1* to chilling and exogenous GA_3_, as well as the presence of the F-box domain in PsF-box1, suggests that it may play a role as an important component of the SCF complex during bud dormancy release. In the SCF complex, the C-terminus of the F-box recognizes specific substrate proteins, and the F-box domain interacts with SKP1 to form the SCF complex [29]. Therefore, we searched for *SKP-*like sequences in the tree peony genome data by local BLAST. In total, 12 *SKP-*like sequences were obtained after redundancy removal, among them four members were differentially expressed during chilling-induced dormancy release [12], and three differentially expressed members were found during exogenous GA_3_-induced dormancy release [10], of which two members were common. One *SKP-*like sequence had an ORF of 537 bp encoding 179 aa (GenBank accession no. JI448135), and another sequence had an ORF of 468 bp encoding 156 aa (GenBank accession no. JI450086) (File SS1, see online supplementary material). The phylogenetic tree of two putative SKP proteins with 22 *Arabidopsis* SKP proteins (ASKs) and SKP proteins of the known plants indicated that one was closely clustered with SsSKP1 (79.74% identity), and it was called PsSKP1 (Fig. S7, see online supplementary material). Another was clustered with AtASK13 (60.53% identity), and it was designed as PsSKP13 (Fig. S7, see online supplementary material). The expression levels of two *PsSKPs* were analysed by qRT-PCR, and they were found to be induced by chilling treatment, and *PsSKP1* reached a peak at 21 days of chilling (Fig. 5A), and *PsSKP13* reached a peak at 28 days of chilling (Fig. S8, see online supplementary material). They also responded to exogenous GA_3_ treatment for 12 h, and *PsSKP1* was induced (Fig. 5B), while *PsSKP13* was inhibited (Fig. S8, see online supplementary material). Therefore, *PsSKP1* was used for further study. To verify the interaction between PsF-box1 and PsSKP1, the sequence of *PsF-box1* was truncated into two fragments according to the site of the F-box domain, pGBKT7-PsF-box1, pGBKT7-PsF-box1^N^, and pGBKT7-PsF-box1^C^ with pGADT7-PsSKP1 were co-transformed into yeast Y2HGold competent cells and screened on SD/−Leu/−Trp/-His/−Ade/x-a-gal medium. The results showed that blue yeast colonies were present on SD/−Leu/−Trp/-His/−Ade/x-a-gal with 200 ng.mL^−1^ AbA after co-transformation of pGBKT7-PsF-box1 and pGBKT7-PsF-box1^N^ with pGADT7-PsSKP1 (Fig. 5C). To confirm this interaction recombinant MBP-PsSKP1 and GST-PsF-box1 vectors were constructed. The GST-PsF-box1 and MBP-PsSKP1 fusion proteins were expressed and isolated. The pull-down results showed that strong signals could be detected after co-incubation with MBP-PsSKP1 and GST-PsF-box1 (Fig. 5D). Taken together, these results indicate that PsF-box1 and PsSKP1 interact, with the specific binding site being the fragment containing the F-box domain.

## Discussion

GA signal transduction is involved in many plant physiological processes, including stem elongation, seed germination, root growth, cell division and elongation, and flowering [30–33]. Recently, GA signals were found to play a vital role during bud dormancy release in perennial woody plants, including apricot, pear, grape, and *Prunus mume * [11, 15, 24, 33, 34]. In tree peony, effective chilling accumulation promotes bud dormancy release and bud break [27], and exogenous GA treatment increases the endogenous gibberellin content and accelerates bud dormancy release [10]. In this study, the content of endogenous active gibberellins increased with the prolonged chilling. The GA content has similarly been found to increase with extension of chilling treatment in poplar buds [35]. Therefore, GA signaling is involved in bud dormancy release in tree peony. In terms of the components of the GA signaling pathway that are involved in bud dormancy release, we found that the PsRGL1 protein levels decreased during chilling- and exogenous GA-induced dormancy release, and that the silencing of *PsRGL1* promoted tree peony bud dormancy release. *PsF-box1* expression was induced by chilling and exogenous GA_3_ treatments, and the overexpression of *PsF-box1* promoted tree peony bud dormancy release. PsF-box1 arrested PsRGL1 and mediated the ubiquitination-dependent degradation of PsRGL1. These results indicate that PsRGL1 is a key component of GA signaling, which negatively regulates bud dormancy release via the ubiquitination-dependent degradation triggered by PsF-box1.

### Main components of SCF^F-box1^ complex during bud dormancy release

Although the GA-GID1-DELLA module and GA-induced degradation of DELLA proteins by SCF^SLY1/GID2^ E3 ubiquitin ligase have been identified in *Arabidopsis* and rice, the GA response mechanisms in different plants are complex because each plant has specifically signal paralogs [17, 18, 21, 36–39]. In this study, we identified a set of final transactors of GA signaling, including DELLA, F-box, and SKP1, during chilling-induced bud dormancy release. GID2 is required for the GA response [40]. In rice, GID2 physically interacts with OsSKP15 and OsCUL1 to form the SCF^GID2^ complex [40], and the level of SLR1 is greatly increased in the *gid2* mutant [21]. In our study, although three F-box family members responded to chilling and exogenous gibberellin treatment, only *PsF-box1* was upregulated, and PsF-box1 protein clustered with the other GID2 proteins (Fig. 4). One *SKP1*-like sequence was identified, and the PsSKP1 protein clustered with AtSKP1 (Fig. S7, see online supplementary material). *PsSKP1* was also upregulated by chilling and exogenous GA_3_ treatments (Fig. 5), thus indicating that it was co-expressed with *PsF-box1*. In *Arabidopsis*, AtSKP1 is involved in the formation of the SCF complex by interaction with several F-box proteins, such as TIR1, COI1, ORE9, EID1, and UFO, and it regulates plant growth and development through a diverse set of signal transduction pathways, including phytochrome A-specific light, auxin, and jasmone signaling, indicating that AtASK1 may be a master component of SCF complexes in plants [41–43]. In this study, the yeast two-hybrid and pull-down results indicated that PsSKP1 can interact with PsF-box1 (Fig. 5), which suggests that they may play a similar role to form the SCF complex. These results revealed the components of the SCF complex during tree peony bud dormancy release, thereby providing insights into the molecular mechanism of GA signaling during tree peony dormancy release.

### PsRGL1 negatively regulates bud dormancy release mediated by PsF-box1

DELLA proteins are key switches in GA signaling pathway. Previous studies have highlighted two possible modes of DELLA protein function: (i) DELLAs act as a transcriptional coactivator [44], and (ii) DELLA interacts with DELLA binding proteins, such as some transcription factors (TF), and inhibits their binding to DNA *cis*-acting element or transcriptional activities [45]. Therefore, it is useful to identify the key DELLA members and their function in bud dormancy release. In *Arabidopsis*, five DELLA proteins possess an N-terminal DELLA domain, VHYNP motifs, a poly (S/T) region, and a C-terminal GRAS function domain [46]. In herbaceous peonies, the expression of *PlDELLA* first increases and reaches its maximum value after chilling for 7 d before decreasing until 35 d and its silencing promotes bud break [47]. In Japanese apricot, the expression of *PmRGL2* is high during ecodormancy [24]. In the present study, we obtained two DELLA members from the tree peony dormant bud transcriptome. However, their transcript levels fluctuated during chilling (Fig. S4, see online supplementary material), and thus the key *DELLA* gene could not be identified at the transcriptional level during chilling- and GA-induced bud dormancy release. We hypothesized that DELLA may function at the protein level. PsRGL1 was subsequently identified as having a downregulated pattern in a cell-free degradation assay, and high levels of polyubiquitination were detected during dormancy release (Fig. 1). *PlDELLA* negatively regulates dormancy release and plant growth in herbaceous peonies, and its overexpression inhibits seed germination and flowering [47] . In this study, the silencing of *PsRGL1* promoted tree peony bud dormancy release, and the dormancy release associated genes, including *PsCYCD, PsEBB1*, *PsEBB3*, *PsBG6*, and *PsBG9* were upregulated, which may accelerate the cell cycle and reopen the transport channels. Taken together, these results indicate that this is the key DELLA member functioning in GA signaling during dormancy release in tree peony.

Next, we investigated whether the ubiquitination-dependent degradation of PsRGL1 was mediated by the F-box of the SCF complex. Among the three PsF-box proteins, only the PsF-box1 protein could bind PsRGL1, as determined by yeast two-hybrid, BiFC, and pull-down assays. The specific binding site was shown to be its C-terminal, indicating that PsF-box1 mediated the ubiquitination-dependent degradation of PsRGL1. Similar results have been previously obtained in rice [21]. In rice, the phosphorylation of SLR1 may be necessary to interact with GID2 [41]. F-box proteins are involved in plant growth and development [48–50]. In Japanese plum, *PslSLY1* is involved in fruit development, and its overexpression in *Arabidopsis* results in pronounced enhancement of germination, stem elongation, and fertility [50]. The GmFBL144, a F-box-like protein, can interact with small heat shock protein (sHSP) to negatively regulate plant drought stress tolerance in soybean [51], and soybean GmFBX176 regulates ABA-mediated response to drought and salt stress [52]. *Dunkelroten Licht 1* (*EID1*), which encodes an F-box protein, functions as a negative regulator of phytochrome A (phyA)-specific light signaling, and regulates photomorphogenesis in seedlings, rosette leaf development, and flowering [53]. In our study, *PsF-box1* was first found to promote bud dormancy release, and genes associated with dormancy release, including *PsCYCD, PsEBB1*, *PsEBB3*, *PsBG6*, and *PsBG9*, in *PsF-box1*-OE buds were significantly induced (Fig. 3). Taken together, our results indicate that *PsRGL1* negatively regulates bud dormancy release, and PsF-box1 protein, which is an important component of the SCF complex, mediating the polyubiquitination-dependent degradation of PsRGL1, thereby ultimately promoting bud dormancy release.

DELLA proteins are the central nexus of GA signaling, and are also at the center of plant phytohormone crosstalk. In this study, we identified the key DELLA member PsRGL1, which is involved in dormancy release in tree peony. Due to a lack of DNA-binding domains, DELLA often integrates with other TFs to block the expression of target genes. Screening for proteins that can interact with PsRGL1 would promote the discovery of downstream genes and metabolic processes that influence dormancy release. Thus, there is ample merit in further clarifying the GA response in accelerating dormancy release by PsRGL1.

## Materials and methods

### Material treatment

Four-year-old *P. suffruticosa* ‘Luhehong’ plants were potted at Qingdao Agriculture University in October 2020. Based on our recent study, 21 d of chilling is adequate for ‘Luhehong’ to break dormancy, and only part of the buds can sprout after chilling for 7 d [27]. The plants were moved to a refrigerated room to undergo chilling treatment (4°C, dark/24 h). After chilling treatment for 0, 7, 14, 21, and 28 d, the apical buds were harvested, immediately frozen in liquid nitrogen, and then stored at −80°C until further use. Three replicates were set up, and each replicate included three plants.

After 7 d of chilling treatment, the plants were transferred to a greenhouse (22°C, light 16 h/dark 24 h), and the apical buds were treated with 500 mg.L^−1^ GA_3_, and distilled water as a control (mock). The apical buds were collected after GA_3_ treatment for 0, 12, 24, 72 h, and stored at −80°C until further use.

### Bioinformatics analysis

The DELLA, F-box, and SKP sequences of tree peony were searched by local BLAST analysis in *P. suffruticosa* genome and transcriptomes were surveyed using BioEdit software [54, 55]. Sequence assembly was performed using DNAMAN 9.0 software. The open reading frames (ORF) were identified using ORF Finder (NCBI) (http://www.ncbi.nlm.nih.gov/). The target sequences of Arabidopsis were downloaded from the TAIR website (https://www.arabidopsis.org/tools/bulk/sequences/index.jsp) and the multiple alignments were performed using ClustalW software with default parameters. A phylogenetic tree was constructed based on the neighbor-joining (NJ) model with 1000 bootstrap replications using MEGA 11.0 software.

### Real-time quantitative RT-PCR (qRT-PCR)

Total RNA was extracted from 100 mg of tree peony buds using a SteadyPure Plant RNA Extraction Kit (Accurate Biotechnology, Hunan, China) according to the manufacturer’s instructions. The first strand of cDNA was synthesized from 1 μg of total RNA using a PrimerScript™ RT Reagent Kit (TaKaRa, Dalian, China). qRT-PCR was performed using a SYBR^®^ Premix Ex Taq™ II Kit (TaKaRa, Dalian, China). All reactions were performed in triplicate. Relative expression levels were analysed according to Livak and Schmittgen [56]. The primers used for qRT-PCR are listed in Table S1 (see online supplementary material).

### Subcellular localization

The open reading frames (ORFs) of *PsRGL1*, *PsF-box1*, *PsF-box2*, and *PsF-box3* without a stop codon were amplified to construct PsRGL1-GFP, PsF-box1-GFP, PsF-box2-GFP, and PsF-box3-GFP fusion constructs. These constructs were then transformed into *Agrobacterium* GV3101 and infected to *Nicotiana benthamiana* leaves. DAPI was used to stain the nucleus, and the fluorescence distribution was observed using a laser confocal microscope (TCP SP8; Leica, Germany). The primers used for the subcellular localization are listed in Table S1 (see online supplementary material).

### Fusion protein expression

MBP-PsRGL1, MBP-PsGAI1, GST-PsF-box1, and MBP-PsSKP1 recombinant plasmids were transformed into *Escherichia coli* BL21 (DE3), and empty GST and MBP vectors were used as controls. The positive clones were induced at 18°C overnight using 1.0 mM IPTG, and centrifuged at 4000 rpm for 15 min. The precipitate was lysed, and the supernatant was passed through a chromatographic column with MBP starch resin and GST agarose gel, respectively, with MBP elution buffer (10 mM maltose, MBP binding buffer) and GST elution buffer, respectively. Then, the protein was collected with an ultrafiltration centrifuge tube and stored at 4°C for subsequent use [57]. The primers used for fusion protein expression are listed in Table S1 (see online supplementary material).

### Cell-free degradation assays

The buds after different chilling and GA_3_ treatments were ground using a high-throughput tissue grinder at 1500 rpm for 2 min. The total proteins were extracted in buffer [50 mM Tris–HCl pH 7.5, 10 mM MgCl_2_, 150 mM NaCl, 0.1% NP-40, 1 mM PMSF, and 1× Protease Inhibitor Cocktail (Roche)], which were stored at −80°C until further use.

A total of 250 ng of purified fusion protein (MBP-PsRGL1 or MBP-PsGAI1) was incubated with 50 μg of total protein (at 22°C for 0, 5, 10, 15, 20, 40, and 60 min), after which 10 μM MG132 (a 26 S protease inhibitor) was added as a control group. The reaction solutions were boiled for 10 min in SDS loading buffer, and then cooled quickly to 0°C for 5 min. The proteins in the reaction solutions were separated by SDS-PAGE gel, and then transferred to a PVDF membrane (Bio-Rad, USA). The MBP antibody (1:5000) was used to incubate with the PVDF membrane, followed by incubation with goat anti-rabbit IgG horseradish peroxidase-conjugated secondary antibody (1:10000). The primers used for the cell-free degradation assays are listed in Table S1 (see online supplementary material).

### Preparation of anti-PsRGL1 antibody and immunoblot analysis

The purified recombinant MBP-PsRGL1 protein band was used to inoculate mice to generate anti-PsRGL1 antibodies (ABclonal, Wuhan, China). The total proteins of buds after chilling (0, 7, 14, 21, and 28 d) were extracted using protein extraction buffer containing 10 μM MG132. Equal amounts of total protein in SDS loading buffer were boiled for 10 min and then cooled quickly to 0°C for 5 min. The proteins were separated by SDS-PAGE and transferred to a PVDF membrane (Bio-Rad, USA). The membrane was incubated with PsRGL1 antibody (1:5000), followed by incubation with goat anti-rabbit IgG horseradish peroxidase-conjugated secondary antibody (1:10000). A plant-specific Actin protein antibody (ABclonal, Wuhan, China) (1: 5000) was used to detect tree peony PsActin protein. The primers used for the immunoblot analysis of PsRGL1 are listed in Table S1 (see online supplementary material).

### Yeast two-hybrid assays

The ORFs of *PsF-box1*, *PsF-box2*, and *PsF-box3* were cloned into pGADT7 vector to generate pGADT7-PsF-box1, pGADT7-PsF-box2, and pGADT7-PsF-box3 vectors. The ORF of *PsF-box1* was truncated into two fragments based on the position of the F-box domain, *PsF-box1^N^* with the F-box domain (1–240 bp) and *PsF-box1^C^* without the F-box domain (241–567 bp), and the pGADT7-PsF-box1^N^ and pGADT7-PsF-box1^C^ vectors were generated. The ORFs of *PsRGL1* and *PsSKP1* were inserted into pGBKT7 to generate the pGBKT7-*PsRGL1* and pGBKT7-*PsSKP1* bait vectors. The BD and AD vectors were co-transformed into yeast Y2HGold competent cells (Clontech, USA), and the positive clones were selected on SD/−Leu/−Trp/-His/−Ade/x-a-gal plates with the corresponding concentration of AbA. The primers used for the yeast two-hybrid are listed in Table S1 (see online supplementary material).

### BiFC assays

The ORFs of *PsF-box1*, *PsF-box1^N^*, and *PsF-box1^C^* were inserted into 35S-SYPCE (CE) vector, and the *PsRGL1* ORF was ligated into 35S-SPYNE173 (NE) vector according to the method described by Ye *et al.* [57]. *Nicotiana benthamiana* leaf after infection for 48 h was used to observe the fluorescence distribution using a laser confocal microscope (TCP SP8; Leica, Germany). The primers used for BiFC are listed in Table S1 (see online supplementary material).

### Pull-down assay

GST or GST-PsF-box1 proteins were incubated with glutathione agarose beads at 4°C for 1 h. The magnetic beads were separated and mixed with 1 mL of PBS buffer before separating. These magnetic beads were incubated with the purified MBP-PsRGL1 protein (or MBP-PsSKP1 protein) at 4°C for 1 h and were separated thereafter. These magnetic beads were added into three-times volume of the elution buffer (Tris–HCl 50 mM, reduced glutathione 10 mM, pH 8.0), and then incubated for 5–10 min, and supernatant was collected. Western blotting was used to analyse the result of pull-down as described by Ye *et al.* [57]. The primers used for the pull-down assays are listed in Table S1 (see online supplementary material).

### Construction of TRV2-*PsRGL1* silencing vector and transformation

Based on the results of alignment of PsRGL1 with other DELLA proteins, the specific fragment of *PsRGL1* was inserted into TRV2 vector to obtain the TRV2-*PsRGL1* recombinant vector as described by Zhang *et al.* [58]. TRV2 and TRV2-*PsRGL1* were mixed with TRV1 at a volume ratio of 1:1 and placed for 4–6 h in a dark room. After chilling for 7 d, the bud scales were removed, and they were sterilized and submerged in infiltration buffer for 3–4 min in a vacuum dryer at 0.3 MPa. The buds were then transferred to 1/2 MS medium containing 200 μM acetosyringone. After 4 d of dark treatment (8°C, 3 d followed by 22°C, 1 d), the buds were transferred into MS medium (200 mg.L^−1^ ticarcillin, and 0.5 M MES) and cultured in an incubator (22°C, 16 h light/8 h dark). After being infected for 10 d, total RNA was extracted, qRT-PCR was performed to analyse the silencing efficiency, and the expression of genes associated with dormancy release, including *PsCYCD*, *PsEBB1*, *PsEBB3*, *PsBG6*, and *PsBG9*, were analysed. Morphological changes, including the relative increase in bud width and height, were measured each day. A total of 60 buds were used per transformation, of which 30 were used for detection of expression and 30 for morphological observation. The data were analysed by one-way analysis of variance (ANOVA) using SPSS 26.0 software, and multiple comparisons were performed using the DUNCAN method, with a significance level of *P* < 0.05.

### Construction of *PsF-box1* overexpression vector and transformation

Super1300-PsF-box1 recombinant plasmid was constructed and transformed into *Agrobacterium* EHA105 (empty Super1300 plasmid as a control). Under sterile conditions, tree peony buds were infiltrated after chilling for 7 d as described by Zhang *et al.* [58]. The buds were immersed in the bacterial solution and infiltrated at a vacuum of 0.7 atm for 5 min, then slowly deflated to allow the bacterial solution to enter the buds. The infiltration step was repeated two times. After releasing the vacuum, the buds were washed for 4–5 times with sterile water before inoculating into MS medium, cultured in the dark for 3 d at 8°C, and then cultured in an incubator (22°C, 16 h light/8 h dark) as described above.

After infiltration for 7 d, the relative expression levels of *PsF-box1* were analysed by qRT-PCR, and those higher than 1.5 times were used to analyse the expression of dormancy release marker genes. The flower bud phenotype (relative growth in bud length and width) was also investigated. The data were analysed by one-way analysis of variance (ANOVA) using SPSS 26.0 software, and the multiple comparisons were performed using the DUNCAN method, with a significance level of *P* < 0.05.

## Acknowledgments

This work was supported by grants from National Natural Science Foundation of China (31872145, 31972452), the Agricultural Seed Engineering Project of Shandong Province (2020LZGC011–1–4), and the National Key R&D Program of China (2018YFD1000403). The funding bodies had no role in the design of the study, the collection, analysis, and interpretation of data, or in writing the manuscript. We would like to thank Editage (www.editage.cn) for English language editing.

## Author contributions

G.S. and Z.Y. conceived and designed the experimental plan. G.L., C.T., and N.D. conducted the experiments. Z.Y. and Y.Y. analysed the data. Z.Y., L.C. and G.S. prepared and revised the manuscript. All authors have reviewed and approved the final manuscript.

## Data availability

The sequence data that supports the findings of this study are available in NCBI and TAIR database with the following accession numbers: PsEBB1 (OP095871), PsEBB3 (OP095872), PsCYCD (OP095873), PsBG6 (OP095874), PsBG9 (OP734236), PsF-box1 (JI545894), PsF-box2 (JI447773), PsF-box3 (JI445105), PsSKP1 (JI448135), PsRGL1 (OP272869), PsGAI1(OP272870), AtGAI (CAA75492.1), AtRGA (Q9SLH3.1), AtRGL1 (OAP14146.1), AtRGL2 (OAP05206.1), AtRGL3 (OAO92748.1).

## Conflict of interest statement

None declared.

## Supplementary data

Supplementary data is available at *Horticulture Research* online.

## Supplementary Material

Web_Material_uhad044
