# Correction to: PsRGL1 negatively regulates chilling- and gibberellin-induced dormancy release by PsF-box1-mediated targeting for proteolytic degradation in tree peony

**DOI:** 10.1093/hr/uhae132

**Published:** 2024-05-07

This is a correction to: Linqiang Gao, Demei Niu, Tianyu Chi, Yanchao Yuan, Chunying Liu, Shupeng Gai, Yuxi Zhang, PsRGL1 negatively regulates chilling- and gibberellin-induced dormancy release by PsF-box1-mediated targeting for proteolytic degradation in tree peony, Horticulture Research, Volume 10, Issue 5, May 2023, uhad044, https://doi.org/10.1093/hr/uhad044.

Since the publication of this article, the authors have noticed errors. Because of a careless mistake in drawing, the cell-free result of PsGAI1 in Figure 1B after exogenous GA3 treatment was pasted in the cell-free result of PsGAI1 in Figure 1A after different chilling-treated days, and the subcellular localization of PsF-box1 was pasted in that of PsF-box2 in Figure 2F. Consequently, corrections are required for two figures. The corresponding figure captions are correct and remain unchanged.

The main purpose of this article is to investigate the interaction between PsRGL1 and PsF-box1 and their functions, and the incorrection of these two parts do not affect the conclusion of this article. In addition, the editor of “Horticulture Research” has reviewed the revised figures. The authors would like to apologize for this error and are now issuing a correction for Fig. 1A and 2F as follows:

**Figure 1:**
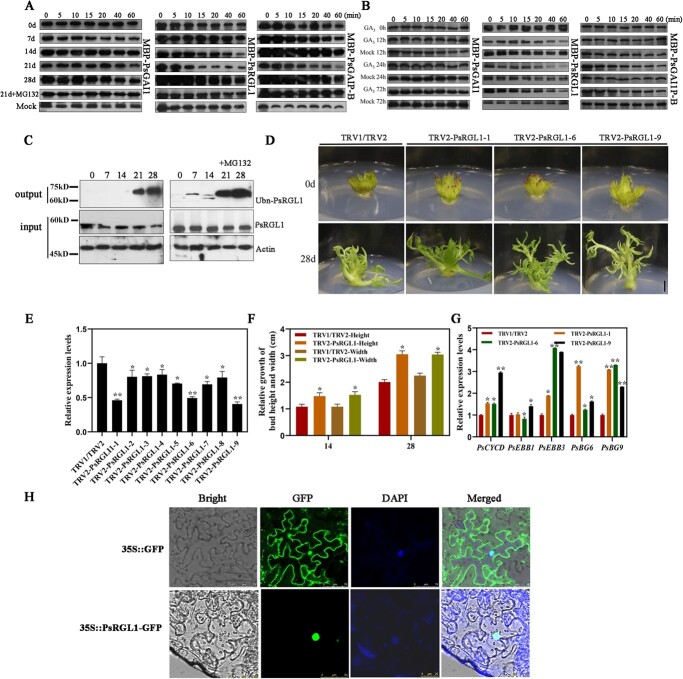
Identification of PsDELLA proteins associated with dormancy release in tree peony. **A** Cell-free degradation assays of three PsDELLAs protein after chilling. The tree peony buds after different chilling-treated days were used to extract total proteins, which were incubated with recombinant MBP-PsRGL1, MBP-PsGAI1, and MBP-PsGAIP-B. 21 d + MG132 (10 μM) represents the total protein of 21 chilling buds incubated with MBP-PsRGL1, MBP-PsGAI1, and MBP-PsGAIP-B containing MG132 (10 μM). Mock represented only recombinant MBP-PsRGL1 and MBP-PsGAI1 without total tree peony protein under incubation conditions, respectively. **B** Cell-free degradation assays of three PsDELLAs protein after exogenous GA_3_ treatment.**C** Polyubiquitination of PsRGL1 during chilling-induced bud dormancy release. The total protein from buds after different chilling days was probed with anti-PsRGL1 antibody, and total ubiquitinated PsRGL1 was detected with anti-Ub antibody in the output. The accumulations of PsRGL1 and actin were tested in the input. **D** Morphological changes of TRV2-*PsRGL1* transgenic buds after being transformed for 28 d. Scale bar, 1.0 cm. **E** Relative expression level of *PsRGL1* in *PsRGL1-*silenced buds by qRT-PCR after being infected for 7 d. **F** Relative expression levels of genes, including *PsCYCD, PsEBB1*, *PsEBB3*, *PsBG6*, and *PsBG9*, associated with tree peony dormancy release by qRT-PCR in *PsRGL1-*silenced buds after being transformed for 7 d.**G** Relative growth in terms of height and width in in *PsRGL1-*silenced buds after being transformed for 14 d and 28 d. Data are represented as the means ± standard deviation (SD) of six replicates. ^*^P < 0.05; ^**^P < 0.01. **H** Subcellular localization of PsRGL1 by fluorescence microscope at an excitation wavelength of 488 nm.

**Figure 2:**
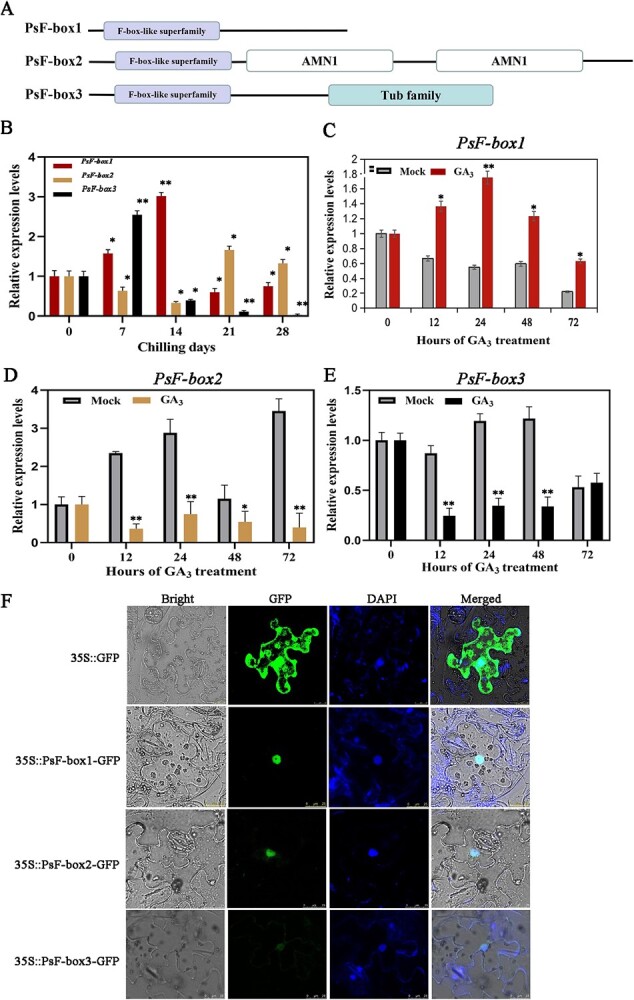
Domain arrangement of the putative PsF-box proteins and the expression patterns of three *PsF-box* genes during chilling- and GA_3_-induced dormancy release. **A** Linear representation of the domain arrangement of the putative PsF-box proteins, of which PsF-box1 had a 567 bp ORF encoding 189 aa, PsF-box2 had a 1617 bp ORF encoding 539 aa, and PsF-box3 had a 777 bp ORF encoding 259 aa. Boxes with different colors represent different domains. **B** Expression patterns of *PsF-box1*, *PsF-box2*, and *PsF-box3* after different chilling durations. **C**–**E** Expression of *PsF-box1*, *PsF-box2*, and *PsF-box3* after exogenous GA_3_ treatment, respectively. **F** Subcellular localization of PsF-box1, PsF-box2, and PsF-box3 by fluorescence microscope with an excitation wavelength of 488 nm. Data represent the mean ± SD of six replicates. ^*^P < 0.05; ^**^P < 0.01.

