# Supplementary material for: PsRGL1 negatively regulates chilling- and gibberellin-induced dormancy release by PsF-box1-mediated targeting for proteolytic degradation in tree peony
Source: Hortic Res. 2023 Mar 13;10(5):uhad044. doi: 10.1093/hr/uhad044 (PMC10541556; doi:10.1093/hr/uhad044)
Supplement: Web_Material_uhad044 [file web_material_uhad044.zip › Supplemental file 1-20230218.docx]

***PsRGL1* (OP272869)**

TCTCAAACCATCTTCCGCTCTCTTTCACCTATCACTTTTCACTCCCCCTGTCCTCCTCCTGTACAAATATCATCCATGCATCTTTTCTCTCTCTAGAATCTGTCCGCCATGTTCACCAATATCCTCCAAGCCTCGCTCCATTTCTGATCACATAATTTCTCAAAGTCCAATCTCTCACATATGGCCACCTACGACCCTGCCATTTCTGCTGCGACCTGCAGCAGTAGTAGCAGCTCGTCATC**ATG**CGCCGCCGGCAAACAAACACCACGCCAAGATTTAGATGGCCTCCTCGCCGGCGCTGGATACAAGGTCCGGTCCTCAGAGCTCCGGCACGTCGCCCGACGTTTGGAGCACCTCGAGACAGCCATGGTCAACGCCCCTTCCGAGATCTCCCAGCTCGCCTCCGACGCTGTACACTACAATCCCTCCGACCTCGCCTCGTGGGTCGACTCACTCCTCACCGAGTTCAGTCAACCGCCTTTACCCTTACCGTCCGATCTATCAGACTTTTCCGATCTTACCTACGCTGATAACTTAGTGGGTCTAGATCAGGCGGCGGTGGATAACGTTTGGACGGACAATTTTACGGCTCAGCAACCGGAAGGACTTCCTCAGCAGTTAACGGCTGGGCCGGCCGTGGAAGAGGATTCCGGCATCAGGTTGGTTCATATGTTGATGACGTGTGCTGAGTCAGTCCAACGTGGCGATCTTCCACTGGCTGGCTCTTTAATCGAAGAGATGCAGGGTTTGTTGACACGTGTAAACACGAGATGTGGGATCGGAAAAGTGGCAGGGTACTTCATCGACGCGCTGAGCCGCCGTCTTTTCGCACCGCACACTGTACCAGTCGGCGGTGGCACGGCCTACGAGAACGAGATTTTGTATCATCATTTTTACGAAGCTTGTCCTTACTTGAAGTTTGCTCACTTCACTGCTAATCAAGCCATCCTCGAAGCATTTGACGGTCACGATTGCGTCCATGTCATCGACTTCAACTTAATGCACGGTTTACAATGGCCGGCACTCATTCAGGCACTTGCTTTACGTCCCGGTGGACCACCTTTGCTCAGGCTAACCGGCATTGGGCCACCATCACCAGACGGTCGTGACTCGCTCCGCGAAATTGGGTTGCGACTCGCCGAGTTAGCTCGGTCTGTGAACGTCCGTTTTGCTTTCCGTGGCGTTGCGGCTACTCGGCTTGAAGATGTGAAACCGTGGATGCTTCAGGTGAGTCCAAAAGAAGCAGTGGCGGTAAACTCAATCATGCAGCTGCACCGATTACTGGGTTCGCCTCCATGCCGCGGCTCTCCGATCGAAACGGTTCTCACTTGGATCCGGAGTTTGTACCCGAAAATCGTGACTGTGGTTGAGCAAGAAGCGAACCACAACCAACCCGAGTTCCTGGACCGGTTCACGGAAGCTTTGTATTACTACTCGACCATGTTCGACTCGCTGGAGGCGTGTTCGATGCAACCGGAGAAATCTGTAGCCGAGATTTACATGCAGAGGGAGATATGTAACGTGCTATGTTGTGAAGGGGCGGCTCGAGTAGAGAGGCATGAGCCGTTGGTAAAGTGGAGGAATCGGCTTGTGGGAGCTGGGTTCAGGTCATTGCATCTGGGGTCGAATGCGTTTAAGCAGGCGAGTATGTTGTTGACGTTGTTCTCGGCGGAAGGGTACTGTGTGGAGGAGAAGGAAGGGTGTTTGACGCTTGGGTGGCATAGCCGGCCACTTATCTCGGCTTCGGCTTGGCAAGCTTCGCCCGATGCAAGTAGAATAATAAATCATAATGTGTTG**TGA**ATTTCGTTGCACGATTTTCTTATTAGAAATGGAAAAAATATTTTATCAATCCGCTTCGTCGTTTCCAAATTTTTCTTCGTTTTCATGGTGGACCTGCCGGAAAGCGAGATTTAGTGTCTGTTATCGTATGTTTATGTAAAAAGCCATAAAAATTAAAATGGAGGTAGTAAAAAAAAAAATAAAATGGAAAATAAATCTTGTCTACTCCTTTGCTTTGCACTTCAA

***PsGAI1* (OP272870)**

GCTGCTTTTAAGTATTAGCCGGAACCCATTTCTTTTATATATTTATATAAACCTCAAAAAATAAAATAAAAAACCATCATTATTATGGTTTCTGCTTTTCCTTATCTCTTCCAGTCTTCAGCTTCCTCCTGTTCCATCTTTGGAGCTCGCGCCTCCCCAAAACCCTATGTTTAATCTTTTCAGTCTTCATTCACCAAAAATGCAGAAAACCAAGCAACCAAACCCCAATCAGATGAACACCCATACCTTCTTTCTCGCTCTTTGATTAGTTGATTGTTTCTGTTTCTTTTTCCTTTTCTGTTTGTCTTTCTTTTTTCAGAATCATCATACCCCAAAAAACAAAAAATGAAGAGAGATCACACCGAGAGTGGTATTTACAGCAAAGGAGAGAGTTCTTCCATGGTGTCTGCAGGGAAGGGAAAGATGTGGGAAGATGACGGGGTACAAGATACAAGCGGCAGCGGCAGCGGCGGAATGG**ATG**AACTTTTGGCTGTTTTGGGTTACAAGGTTAAGTCTTCAGACATGGCGGATGTGGCTCAGAAGCTTGAACAGCTCGAGATGGTCATGGGCACAGCTCAAGAAGATGGAATTTCCCATCTTTCCTCTGACATGGTTCACTACAACCCCTCAGATATTTCTGGGTGGCTGGGAAGTATGCTTTCTGAGTTCAACGCTCCTCCGATTGACACTGGTGATGATTTCTTCGATTCTTGTTCTGCAGTTCTCAATGACCAGATGATGAATCAAACCGACGATGGTCTTTCTTTCGCCTCTGCTCCTGGTAACTCCTCTTCAATGTCAAGCATCAATTTCTCAGGGCAGCAGCAGCAGCCATCGTTTGCGACGTTTCAAGACCATTCCCAGTATGACCTGAGTGCGATTCCCGGTGTTGCTGTTTACCCACAAAAGAGAGAATCACCCGAGCTGCAAAATGGCTGGAAGCGAATGAAGATGACGATGGGATCTAATCCAAACCCAATGATGATACCGTCCCCTGTGTCATCAACCACCACCGTTGCTGGTTTGAATAATGCTTCGGTCGACTCAGCTCGTCATGTTGTGCTGGACTCACAGGAAACCGGTGTTCGACTCGTTCACACCTTGATGGCTTGTGGGGAGGCTGTTCAGCAAGATAATCTCAAGTTGGCTGATGCCCTGGTGAAACACATCGGTTTGCTTGCAGCGTCCCAAGCTGGCGCGATGAGGAAGGTCGCGACGTATTTCGCCGAAGCATTGGCTCGAAGAATTTACAGAATTTACCCACAAGACTGTCTCGATTCATCCTACTCCGATATCCTCCAGATGCACTTCTATGAAACCTGCCCCTACCTCAAATTCGCCCATTTCACAGCGAATCAAGCAATACTCGAAGCTTTTGGGAGTGCCACCAGAGTTCATGTGATCGATTTTGGGCTGAAACAGGGGATGCAATGGCCTGCGTTGATGCAAGCTCTGGCATTACGTCCCGGCGGGCCTCCGGCCTTTCGTCTCACCGGAATTGGCCCACCGCAGGCGGATAACACCGATGCTTTGCAGCAAGTGGGGTGGAAGCTTGCCCAATTGGCTGAAACAATTGGCGTTGAGTTTGAATTCCGTGGATTCGTTGCCAATAGTTTAGCAGATCTCGAGCCGGCTATGCTCAACATCCGGCCACCGGAGGTGGAAGTTGTGGCGGTGAACGCTGTCTTTGAGCTCCATCGTCTCCTAGCTCGACCGGGTGCAATCGAAAAGGTGCTCTCCTCAATCAAGGCAATGAAGCCAAAAATTGTCACCATCGTGGAACAAGAAGCCAACCACAACGGTCCTGTTTTCTTAGACAGGTTCAACGAGGCTCTGCATTACTACTCGAACTTGTTCGACTCGTTGGAGGGGTCTGGGTTTGCACCGCCGAATCAGGACCTGGTCATGTCCGAGATTTATCTGGGTCGGCAGATTTGTAACGTGGTGGCTTGTGAAGGGGCTGAGCGAGTTGAGCGACATGAGACATTGACTCAGTGGCGGGATCGGATGACTTCAGGTGGGTTCGACCCGGTTCACTTGGGGTCCAACGCGTTCAAGCAGGCGAGTATGCTCTTGGCTTTGTTTGCAGGTGGGGATGGGTACATGGTGGATGAAAGCAATGGGTGTCTCATGCTTGGGTGGCACACAAGGCCGCTCATCGCCACATCCGCCTGGCAGCTCGCCGCCAACGGTGTTCCAGAGGAG**TAA**TGTGTTTCGGGAATCCAGTAGATCGAGTCAACTCAATAACTTTGGACGAGTCGTATGTTAAGTTTGGTTTAATTAGATTTGTGAGAGTGAGGCTATAGAGTTGAAACGGGATGAAGTGCGTAACTCTTCCTTTTTTTTCGTTTTTACCTCTCCAAGTCCAAGTTAGCCCTTTTTCTATCTTTTTGCCTTTTTATCTTGGAACTTTTAATTAGCCTTTCTAAATATTTGAGAGGTTAATCAATGCGAATGTGTGGATGTCTATATTTTGACTTTCTGAATGAGCAAAAGGATTATCTTAAAAAAAAAAAAAAA

***PsGAIP-B* (OQ224301)**

ACAGAATTAGTTAATTGGAATTTTATTTTAATTAATTACATAAAACTAAATTCAATTCCTTACACTACTTAAACACCAGGTAAGCAAAATGGTGCAAACATATCGTGCCGCACTCCATTGTACCCTTTTCCCCACAAAGAAAATGAAATGAAATTTTCACATAAAAGATGTTATTTCCACCTTATCTGTTTGAGCTGTAGTCGTTGTTGTTTGCCTGTTGTTTCATTGGTGGTCGCGCCAAGTCACGAGTGTTTCTTATTATTATTTTCTCTCTCCTTCTGATGCTGCAGAACTAGTACAACTCGAGTGGTGAAAAACCAAGCAAGAAAACCCAATCCCCAATGCCCAGATTCTTAATCCATTCATCGTCCCCTTTGATTTCCGAAACTCCCCAGACGAACACACGCACATACACATAAAAAAACCCAGCCTAATCCAGAACCAATCAAAAAT**ATG**AAGAGAGACCACCATAGTCTTCAACCTCGCCCAGATTCCACCATTAGCGGTGGTGGCAGCAGCAACTCTAACGGCGGTTGTGTTGGTGGTTATTCAATGGCAGCTGCCGGAAAATCGAAGATGTGGGTAGACGAAGGTCAACAAGATGCCGGCGTAGACGAGCTTTTGGCGGTTTTGGGATATAAGGTCAGGTCATCGGACATGGCAGATGTAGCTCAAAAGCTTGAGCAACTCGAAGAGGCCATGGGTACTGTTCAAGAGGATGGTCTCTCACATATCGCTTCCGAGACGGTCCATTACAACCCATCTGACATGTCCAACTGGCTCGAGAGTATGCTGTCCGAGTTCAACCCGCCTAATATCGATCATTCTCTTCAACCTCTCCCGCAATCCATTGACGATAATTCGTTCCTTGCTCCTGCCGAATCGTCCACCATCTCATCGGTGAGTTTCTCTGGCCATCTAAATCAACAGCAGCAGCAACACCGTAATGAGCATCGTCAACCCTTTCAAGACTCGTCTTCTTCTGTTTACGGTCTGAAAGGTATTCCAGGTAAAGCACTCTACACACAGATCGAATCTCCCATCCGAGAGGCCAAACGATTGAAACCGTCAATCCCAACGGTATCGTCCTCAGCATCCTCGAATGTTGGGATGTGGGGAGGAGTCACCGCTGAGTCCAAGACGGCGACTCGGCCTGTAGTCCTAGTCGATTCACAAGACACCGGAATCCGACTTGTGCACAGCATGATGGCCTGCGCCGAAGCAATTCAAAACAACAGTCTGAATCTAGCGGAAACTCTATTGAAGCAGATCGGTGCCCTCGCAGCCTCGCAGGCCGGTGCCATGCGAAAGGTCGCTACATACTTCGCTGAAGCACTAGCGCGTCGAATTTACCGATTGTATCCTCAAGCATCTCTCGATCCATCCGTCTCCGACCTCCTCCAGATGCACTTTTACGAAACCTGTCCGTATCTCAAATTCGCGCACTTCACAGCGAATCAAGCCATTCTAGAAGCCTTCGCCGGCAAAAAGCGCGTTCATGTCATCGATTTCAGTGTCAATCAAGGTATGCAGTGGCCGGCGCTTATGCAAGCTCTGGCGTTGCGTCAAGGTGGCCCTCCCGCATTCCGATTAACCGGAATTGGTCCTCCGTCGTCGGACAACACCGATCATCTTCAGGAGGTGGGTTGGAAACTCGCTCAGTTCGCCGAGACGATTCACGTCGAGTTTGAATACAGAGGCTTCGTGGCCAACAGTTTGGCGGATCTTGACGCGTCGCTGCTCGATCTCAGACCAAGTGAGGTCGAGGCGGTGGCGGTTAATTCGGTATTCGAGTTGCACAAATTGCTGGCTCGGCCCGGAGCAATGGAAAGGGTGTTGACGGTGGTGAAGGAGTTGAAACCGGTGATAATGACCGTCGTGGAACAGGAAGCGAACCATAACAGTCCGGTGTTCCTAGACCGGTTCACCGAGTCGCTCCATTACTACTCGACAATGTTTGACTCGTTAGAAGGATCGGCAAGTGGCGAAGACAAGATGATGTCAGAGGTATATTTAGGGCGGCAGATATGCAACGTGGTGGCGTGTGAGGGAGCGGACCGGGATGAACGGCACGAGACACTGGGTCAGTGGCGATCAAGGTTAGGCTCGTCCGGGTTCGAGCCGGTTCACCTGGGGTCCAATGCGTTCAAGCAAGCGAGCATGCTATTGGCTGTGTACGGCGGTGGAGATGGGTATAGGGTGGAAGAAAAGAATGGGTGCCTCATGCTTGGATGGCACACTCGTCCACTCATTGCCACCTCGGCTTGGCAACTCAGTTGTAAAGCAAGACCTACCCAC**TGA**GTCAGCTCAACCAACCCACCAATCAAATCCCTGACCCCTCCAAAAAAAAGAATAACCATTCCCCTAACCCGGTGCCAGTAGTATGATGCGTTCTTTCTGGGTCATGGGTGAGACCAATCAGTGAGACTTGAGACCCAC

***PsF-box1* (JI545895)**

ACATGGGAACCAACAAGTTTCTGCATATAGCTTCAGAACCTTGTAATTTCCTTGATCCACAGATGAAGCGATCACACTCCGGCGACGATGTTCCGGCTATCGGAGAGGACAAGAAG**ATG**AAGAAGATAAAGGTTTCAGAAGAGGAAGAAGAAGAAAAAGAAGAGAACTTTATGCTTTTTGACGAGAATTTACTTTACGAGGTGCTCAAGCACGCAGACCCGAGGACGGTGGCCACGGCGGCGTGCGTCAGCAAGCAGTGGCACAAGACGGCGCAGGACGAGCGTCTTTGGGAGCTGATCTGTACGAAACACTGGGCTAATATCGGTTGCGGCAACCAACAACTCCGATCCGTGGTTCTTGCTCTTGGTGGGTTTCGTCGTCTCCACTCGCAGTACCTTTGGCCTCTGTCAAAGCCGTCTTCCTCATCTTCATCCACCTCGTCGCCGTCGGCATGGGCTTCGAGTTCTTTGTCACCTACGTTTCCTCCGATGATTGCTTCGTCGTCGTTGTCATCCAAGGCACCATCCAAGGCACCTACTAGATGGGGAAAGGACGAGGTTCAGCTCTCGCTGTCTCTTCTCTCAATTCGTTACTATGAGAAGA**TGA**ATTTCACCAACAGGGGAAGATGAATTTGATCAACAGAGGAATATGAATTTCAGAACAGGGGCAGTTAAATCAAGTCTTTTATTTCGTTCTTTGTTTTAATTCAGCTGTGTAATCTTTGCTTTTTAGTGTATGCTATCTAGGTTTTAACAACTAAGATCTGTAAGCTTATTATGGATTATCAATTTCTATGTTTGTTACGAAAAAAAAAAAAAAAAAAAAAAAAAAAAA

***PsF-box2* (JI447773)**

ATTCGAGCTCGGTACCCGGGGATCCTCTAGAGATTCTAATACGACTCACTATAGGGCAAGCAGTGGTATCAACGCAGAGTACATGGGGACGTCCGTCTCCGAACTTTTCGACGGATCAACGAGGCTCCCCAGGCTCAGGGATTATAGCTCCATTTCTGATTCTCTGGTGCCTATGATAAGGCGGTGAGGATTGCGTTCATATTCCGGCCACCGTGG**ATG**GGCCAGTCAGCCTCCACTGCCGCTGTCCCATCAGGCTCAAGCCGCCGTGACATCAATCATTCCCACCGTTCGAAGAACAAGTTAACGGATCCGATCTATCCGATGGACGACGAAGAAAAGGACGAGTCTCACTGGGCCATCGCCGATGTAGCGTTTGACTATATCTCCGAGTTACCCGACGAGTGTTTGGCTTGCATTTTCCATTTCCTTGGATCCGGTGATCGGAAACAATGTTCGCTCGTTTGCCATCGATGGCTTCAGATCGAGGGACAGAGCCGTCACCGTCTCTCCCTCAACGCCAAATATGATCTGCTTTCCATGATCCCTTCCCTCTTTTCTCGATTCGATTCGGTCACAAAACTCGCTTTGAAATGCGACCGTAAATCGGTAAGCATCGGAGACGATGCTATCACGCTCATATCGCAACGGTGCCGAAATCTCACTCGCCTCAAGCTCCGTGCTTGTCGTGAATTGACCGATGCCGGCATGGAGGCTTTTGCTAAGAATTGCAAGGGTTTGAAGAAGCTATCTTGTGGATCATGCACCTTTGGGGCCAAGGGGATGAACGCCGTGTTCGATCATTGCTCAGCTCTTGAGGACTTATCGGTGAAGCGATTACGAGGCCTTACCGATGGGGCAGCTGCTGAGCCGATTGGACCCGGACTAGCAGCATCGTCTCTCAAAACCATATGCTTGAAGGAACTTTACAACGGCCAGTGTTTCGGGCCGCTTATTACCGGTTCGAAGAATCTTCGAACACTAAAGCTCTTCAGGTGCTCAGGCGATTGGGATAAGCTTCTCCAAGTTATCGCAGATCGAGTTACCAGTATGGTGGAAATTCATCTCGAGAGGATTCAGGTCAGCGATATTGGGCTTGCGGCGATCTCTAAATGCCAGGGTCTCGAGATTTTACATCTTGTCAAGACGCCCGAGTGCACTAATAGTGGGCTTGTGTCCGTCGCGGAACGTTGCAAGCTCTTACGGAAGCTTCACATTGATGGGTGGAAGGCAAATCGTATAGGCGACGAAGGCTTAATGGCTGTTGCTAAATATTGCCCTAACCTTCAAGAACTGGTCCTCATCGGTGTGAATCCCACGAAGTTGAGTCTGGATATGCTTGCATCAAATTGTCAGAATCTAGAGCGGCTGGCATTATGTGGGAGTGAGACAGTTGGCGATGCAGAAATCACATGCATTGCTGCTAAATGCGTTGCTTTGAAGAAGCTATGTATCAAGAGCTGCCCCGTTTCTGATAGCGGAATGGAAGCACTTGCAGGTGGTTGCCCTAATTTGGTAAAGGTGAAGGTGAAGAAGTGCAAGGCAGTAACTTGTGGTGGTGCAGATTGGTCGAGGTCAAGTAGGGCATCACTTGCAGTAAATTTAGACTCTGGAGAACCTGAACATCAGGATGCAAATGGTAGTGATGGTGGAGCCCAGGAAAACGGTGTTGAGTTCCCACCTGTGGGAAATCAAATTGCTGCTGCTAACACTGCATCGAGCAGCACAGGTCGATCATCATCCTTTAAGGCAAGGTTAGGTATTTTAACTGGAAGGAATTTAGTGGCCTGCACTCTAAGAAGGTGGTCAAGCTTTAACAACAATTCACGCCAG**TAA**TTAGAAGTTGAGCTATAAGAGAAACTTGGAGAAAGAAAATTCTCCTGGTTTGTTTACATTACGTTTTCTTGTATTGTCCGTTTTGCATGTTTCATGTTAGTTTTCTATTTATCTTCTTTCTTGTATTTTAAGTTGTGTGCTATTTATTGTCTTTGAAATTGACTTTCACCATTTGGAATTATGATTACAAGGTTGCTTTGTTTATTCAAAAAAAAAAAAAAAAAAAAAAAAAAA

***PsF-box3* (JI445105)**

CCTAATAACGAATTTGCTTTTGACATATTAGAGAAGAATCCGTGGTGAACCCTTATCAGATATTTTTGTGGTTGAAGCTTTAGAGTTCTCTTTGTCGGTGTTAATAGCATGGGTGTATGGATAATTTGATATTTGGGGCATTCCTTGAACGTGGGTTGATAAGAGTTTGGAGGTTGGAGGTTCCTAAATACATTATTTATCAGATTTGAAGTGTAGATAGGCAAGGAACTATCA**ATG**TCTATTAAGAGTATAATTCAGGACATGAAGGGTGAGTTTGGGAACATTTCCAGAAAGGGGTTTGATGTCAAGTTTGGTTATGGATTGAGATCAAAGTCCCATCGGGTGGTTCAGGATTGCTCAATGGTGATTGATGCTTTGAGGCAGAGTTGTTGGGCCAACATGCCGCCAGAGCTTTTGAGGGATGTTCTTATGAGGATTGAGGCATCTGAGGGTTCATGGCCGCCTCGAAAAAATGTGGTTTCATGTGCTGGTGTTTGCAGGAGTTGGAGAGAGATAACTAAAGAAATTGTGAAAATTCCAGAAGTTTCGGGCAAGTTGACCTTCCCTATTTCCTTGAAACAGCCTGGTCCAAGAGACTCTCTTATCCAGTGTTATATAAAAAGAAATCGCAGCAATCAAACATATCATCTTTACCTTGGCCTAAATCAAGCTTCAAATGATGATGGAAAGTTTCTTCTTGCTGCACGGAAGTGTCGAAAAGCTACTTGCACAGATTACATTATCTCTCTAAATGCCGATGATGTGTCAAAGGGGAGCAACAGTTACATTGGGAAATTGAGATCAAATTTTCTGGGGACCAAGTTCACAATCTTTGATGCACAGCCACCTAATGCTGGGGCCAAAGTTACTAAATGTCGTTCTACTAGATTGGTGGGTTCGAAACAAGTCTCCCCCAGACTCCCTGCTGGCAACTATCCTGTAGCCCATGTCGCTTATGAATTGAATGTGTTGGGTTCTAGGGGTCCAAGGAGAATGCAGTGTAACATG**TGA**

***PsSKP1* (JI448135)**

AATTCAGTTCTCGATTTTGTCAAGGGTTAGGGTTTCGTCTTCGTTCTCTTTCATTTTCCGAGAC**ATG**GTTAGGGTCGTGACTTTAAAGAGCTCCGATGGCGAGGTCTTCGAAGTTGATGAGATAGTAGCCCTTGAATCGCAGACGATCAAACACATGATCGAGGATGATTGTGCTGATAACGGCATTCCTTTGCCGAACGTTACCAGCAATATCTTGTCCAAGGTCATTGAATACTGTAAGAAGCACGTGGAGACTCGAAAGCAGTTGGAGACTCTACACGATTTTTATTTGAGAAAAGGTTTTCGAGATGATGTATCGACTATTGCAATTATCTCCTTGGAGGGTCGTAGCGCTGTAGAGGAACTCAAGAATTGGGACACCGAGTTCGTCAAGGTTGATAAGGCGACTCTCTTCGATCTGATTTTGGCTGCGAACTATTTGAACATCAAGAACCTGTTGGACCTTACAAGCCAGGCAGTGGCTGACATGATCAAGGGCATGACGCCAGAAGATATTCGGAAGTTTTTCAACATCAAGAACGAGTTTACCCCCGAGGAGGAAGAGCAGGTTCGTCGGGAGAACCAGTGGGCATTTGAG**TGA**GGTGAACTTTGGAGAAAGTTGTTTATGGTTTGTTAGTCTTATTATAATTATCTATGTGGTGTGTTCTTTATGTTAGCGTTAGTTTGTTTCTACTTTATTTTCATTTTCAAGATTTGGAAACTTCTTTCTGAATCTGGTTGAACTTTGAAAATGTAAATAATGCTTGGATCCCTCTACGTGGATTCAATTGTGCTGTGGTTCGATCTATACTAATGTCACCAAAGTTTAACCC

***PsSKP2* (JI450086)**

ATCATTCCAAGCGTGATACGATTCTTGGTTTCTCACCCAAAAGAGCGAGGGTTAAGTTCTTTTCTCGTTTCCGTTTCCGTTTCCGTTTTCAATC**ATG**TCGTCGTCCAAGACTATTACTCTGAAGAGCTCTGATGGCGAGAAATTCGAAATCGATGAGTCCGTCGCAAACCTATCTCAGACGATCAAACACATGATCGAGGACGAATGTGCCGATAATGGAATCCCGCTGCCCAATGTCACCAGCCAGACCTTGTGTAAGGTCATTGAGTTCTGTAAGAAGCACGTCGAGCCTCCCATGCCTGAAGATCACAAGGCTCAGGAGGATCTCAAGGAGTGGGACAAGGAATTTTGCAATGTTGATCAGGCCGTTCTCTATGATCTCCTTTTGGCTGCGAATTATCTCAATGTGAAAGAATTGCTCGATCTGTGTTGCCAAACTGTAGCGGACATCATCAAGGGGAAGACTCCACAAGAGATTCGTGACTATTTTCACATCAAGAATGACTTTACCCCTGATGAGGAGGAAGAAATAAGGAAGGAGAATCAGTGGGCATTCGAG**TAG**AAAGGAAATGCCTTGGTGGAGAAAAAAGTTTAGGAAGTTAAATATTTTTCTTATTATTGTTTGAAGTTTAGTTTCGACTTGGATTTGAGTAAATCGTC
