# Supplementary material for: PsRGL1 negatively regulates chilling- and gibberellin-induced dormancy release by PsF-box1-mediated targeting for proteolytic degradation in tree peony
Source: Hortic Res. 2023 Mar 13;10(5):uhad044. doi: 10.1093/hr/uhad044 (PMC10541556; doi:10.1093/hr/uhad044)
Supplement: Web_Material_uhad044 [file web_material_uhad044.zip › Table S1 The primer information used in this study0218.docx]

**Table S1 The primer information used in this study**

| **Name** | **Sequence (5’-3’)** | **purpose** |
| --- | --- | --- |
| PsF-box1-real-F | TGGGCTAATATCGGTTGCGG | real-time quantitative RT-PCR |
| PsF-box1-real-R | CCTTGGATGGTGCCTTGGAT |  |
| PsActin-real-F | GAGAGATTCCGTTGCCCTGA |  |
| PsActin-real-R | CTCAGGAGGAGCAACCACC |  |
| PsCYCD-real-F | GAGGCCGTGGATTGGATTCT |  |
| PsCYCD-real-R | AAAAGGGGCACTTGGGTCTC |  |
| PsEBB1-real-F | AATAGCCCGCGAAGTCCAAA |  |
| PsEBB1-real-R | GGGATCTGATGAACCAGCCC |  |
| PsEBB3-real-F | GGTGAGATTACTCCGCCACC |  |
| PsEBB3-real-R | CGACCCTGAATCTGAGACCG |  |
| PsBG6-real-F | CCTACTACCCGGCCACAAAG |  |
| PsBG6-real-R | CTACTGAAAGCACCCGCAGA |  |
| PsRGL1-real-F | ACCACGCCAAGATTTAGATG |  |
| PsRGL1-real-R | GTTGACTGAACTCGGTGAGG |  |
| PsGAI-real-F | ATGGTCTTTCTTTCGCCTCT |  |
| PsGAI-real-R | TCATCTTCATTCGCTTCCAG |  |
| PsGAIP-B-real-F | GCCCAGATTCCACCATTAG |  |
| PsGAIP-B-real-R | GAGACCATCCTCTTGAACAGTA |  |
| PsF-box2-real-F | ACGTTGCAAGCTCTTACGGA |  |
| PsF-box2-real-R | TTCTGCATCGCCAACTGTCT |  |
| PsF-box3-real-F | ATGACCCGTAGAGACTGGCT |  |
| PsF-box3-real-R | GCCGGAGTTACCTTGACACA |  |
| PsSKP1-real-F | TCCTTTGCCGAACGTTACCA |  |
| PsSKP1-real-R | ACCTTGACGAACTCGGTGTC |  |
| PsSKP13-real-F | ATGTCACCAGCCAGACCTTG |  |
| PsSKP13-real-R | ATGTCCGCTACAGTTTGGCA |  |
| PsF-box1-GFP-F | atacaccaaatcgactctagaATGAAGCGATCACACTCCGG  (*Xba*I) | Subcellular location |
| PsF-box1-GFP-R | gcccttgctcaccatggtaccTCATCTTCCCCTGTTGGTGAA  (*Kpn*I) |  |
| PsRGL1-GFP-F | atacaccaaatcgactctagaATGGCCACCTACGACCCTG  (*Xba*I) |  |
| PsRGL1-GFP-R | tatttaaatgtcgaccccgggCAACACATTATGATTTATTATTCTACTTGCA  (*Sma*I) |  |
| PsGAI-GFP-F | atacaccaaatcgactctagaATGAAGAGAGATCACACCGAGAGTG  (*Xba*I) |  |
| PsGAI-GFP-R | tatttaaatgtcgaccccgggCTGGATTCCCGAAACACATTACT  (*Sma*I) |  |
| PsF-box2-GFP-F | atacaccaaatcgactctagaATGGGCCAGTCAGCCTCC  (*Xba*I) |  |
| PsF-box2-GFP-R | gcccttgctcaccatggtaccCTGGCGTGAATTGTTGTTAAAGC  (*Kpn*I) |  |
| PsF-box3-GFP-F | atacaccaaatcgactctagaATGGAACATCCGATCTCCTCG  (*Xba*I) |  |
| PsF-box3-GFP-R | gcccttgctcaccatggtaccCCTCCCCCTCTTTGAGCTACA  (*Kpn*I) |  |
| PsRGL1-TRV2-F | agaaggcctccatggggatccCAACCGCCTTTACCCTTACCG  (*BamH*I) | VIGS |
| PsRGL1-TRV2-R | gagacgcgtgagctcggtaccTCTCGTGTTTACACGTGTCAACAA  (*Kpn*I) |  |
| PsRGL1-BDF | cagaggaggacctgcatatgATGGCCACCTACGACCCTG  (*Nde*I) | yeast two-hybrid assays |
| PsRGL1-BDR | cgacggatccccgggaattcTCACAACACATTATGATTTATTATTCTACTTG  (*Eco*RI) |  |
| PsGAI-BDF | cagaggaggacctgcatatgATGAAGAGAGATCACACCGAGAGTG  (*Nde*I) |  |
| PsGAI-BDR | cgacggatccccgggaattcTTACTCCTCTGGAACACCGTTGG  (*Eco*RI) |  |
| PsSKP1-BD-F | cagaggaggacctgcatatgATGTCAGAAGTTAGTATGGCAGTGATC  (*Nde*I) |  |
| PsSKP1-BD-R | cgacggatccccgggaattcTCATTTTCGATCTGGGACTGGT  (*Eco*RI) |  |
| PsSKP1-AD-F | gtaccagattacgctcatatgATGTCAGAAGTTAGTATGGCAGTGATC  (*Nde*I) |  |
| PsSKP1-AD-R | atgcccacccgggtggaattcTCATTTTCGATCTGGGACTGGT  (*Eco*RI) |  |
| PsF-box1-AD-F | gtaccagattacgctcatatgATGAAGCGATCACACTCCGG  (*Nde*I) |  |
| PsF-box1-AD-R | atgcccacccgggtggaattcTCATCTTCCCCTGTTGGTGAA  (*Eco*RI) |  |
| PsF-box1-BD-F | acagaggaggacctgcatatgATGAAGCGATCACACTCCGG  (*Nde*I) |  |
| PsF-box1-BD-R | tcgacggatccccgggaattcTCATCTTCCCCTGTTGGTGAA  (*Eco*RI) |  |
| PsF-box1^C^-AD-F | gtaccagattacgctcatatgACGAAACACTGGGCTAATATCGG  (*Nde*I) |  |
| PsF-box1^C^-AD-R | atgcccacccgggtggaattcTCATCTTCCCCTGTTGGTGAA  (*Eco*RI) |  |
| PsF-box1^N^-AD-F | gtaccagattacgctcatatgATGAAGCGATCACACTCCGG  (*Nde*I) |  |
| PsF-box1^N^-AD-R | atgcccacccgggtggaattcACAGATCAGCTCCCAAAGACGC  (*Eco*RI) |  |
| PsF-box2-AD-F | gtaccagattacgctcatatgATGGGCCAGTCAGCCTCC  (*Nde*I) |  |
| PsF-box2-AD-R | gtaccagattacgctcatatgCTGGCGTGAATTGTTGTTAAAGC  (*Eco*RI) |  |
| PsF-box3-AD-F | gtaccagattacgctcatatgATGGAACATCCGATCTCCTCG  (*Nde*I) |  |
| PsF-box3-AD-R | atgcccacccgggtggaattcCCTCCCCCTCTTTGAGCTACA  (*Eco*RI) |  |
| PsF-box1-pSuper1300-F | atacaccaaatcgactctagaATGAAGCGATCACACTCCGG  (*Xba*I) | *PsGID2* over-expression |
| PsF-box1-pSuper1300-R | gcccttgctcaccatggtaccTCATCTTCCCCTGTTGGTGAA  (*Kpn*I) |  |
| PsSKP1-CE-F | gagaacacgggggactctagaATGGTTAGGGTCGTGACTTTAAAGA  (*Xba*I) | BiFC |
| PsSKP1-CE-R | actatcgatggatccactagtCTCAAATGCCCACTGGTTCTCC  (*Spe*I) |  |
| PsSKP1-NE-F | accgggctcaggcctggcgcgccATGGTTAGGGTCGTGACTTTAAAGA  (*Asc*I) |  |
| PsSKP1-NE-R | cccgggagcggtaccctcgagCTCAAATGCCCACTGGTTCTCC  （*Xho*I） |  |
| PsGAI-NE-F | accgggctcaggcctggcgcgccATGAAGAGAGATCACACCGAGAGTG  (*Asc*I) |  |
| PsGAI-NE-R | cccgggagcggtaccctcgagCTGGATTCCCGAAACACATTACT  （*Xho*I） |  |
| PsGAI-CE-F | gagaacacgggggactctagaATGAAGAGAGATCACACCGAGAGTG  (*Xba*I) |  |
| PsGAI-CE-R | actatcgatggatccactagtCTGGATTCCCGAAACACATTACT  (*Spe*I) |  |
| PsRGL1-NE-F | accgggctcaggcctggcgcgccATGGCCACCTACGACCCTG  (*Asc*I) |  |
| PsRGL1-NE-R | cccgggagcggtaccctcgagCAACACATTATGATTTATTATTCTACTTGCA  （*Xho*I） |  |
| PsRGL1-CE-F | gagaacacgggggactctagaATGGCCACCTACGACCCTG  (*Xba*I) |  |
| PsRGL1-CE-R | actatcgatggatccactagtCAACACATTATGATTTATTATTCTACTTGCA  (*Spe*I) |  |
| PsF-box12-NE-F | accgggctcaggcctggcgcgccATGAAGCGATCACACTCCGG  (*Asc*I) |  |
| PsF-box12-NE-R | cccgggagcggtaccctcgagTCTTCCCCTGTTGGTGAAATTC  (*Xho*I） |  |
| PsF-box1-CE-F | gagaacacgggggactctagaATGAAGCGATCACACTCCGG  (*Xba*I) |  |
| PsF-box1-CE-R | actatcgatggatccactagtTCTTCCCCTGTTGGTGAAATTC  (*Spe*I) |  |
| PsF-box1^C^-CE-F | gagaacacgggggactctagaACGAAACACTGGGCTAATATCGG  (*Xba*I) |  |
| PsF-box1^C^-CE-R | actatcgatggatccactagtTCATCTTCCCCTGTTGGTGAA  (*Spe*I) |  |
| PsF-box1^C^-NE-F | accgggctcaggcctggcgcgccACGAAACACTGGGCTAATATCGG  (*Asc*I) |  |
| PsF-box1^C^-NE-R | cccgggagcggtaccctcgagTCATCTTCCCCTGTTGGTGAA  （*Xho*I） |  |
| GST-PsF-box1-F | ccgcgtggatccccggaattcATGAAGCGATCACACTCCGG  (*EcoR*I) | Fusion protein expression |
| GST-PsF-box1-R | gtcacgatgcggccgctcgagTCTTCCCCTGTTGGTGAAATTC  (*Xba*I) |  |
| MBP-PsSKP1-F | gagggaaggatttcacatatgATGGTTAGGGTCGTGACTTTAAAGA  （*Nde*I) |  |
| MBP-PsSKP1-R | ttaattacctgcagggaattcCTCAAATGCCCACTGGTTCTCC  (*EcoR*I) |  |
| MBP-PsF-box1-F | gagggaaggatttcacatatgATGAAGCGATCACACTCCGG  (*Nde*I) |  |
| MBP-PsF-box1-R | ttaattacctgcagggaattcTCTTCCCCTGTTGGTGAAATTC  (*EcoR*I) |  |
| MBP-PsGAI-F | gagggaaggatttcacatatgATGAAGAGAGATCACACCGAGAGTG  (*Nde*I) |  |
| MBP-PsGAI-R | ttaattacctgcagggaattcCTCCTCTGGAACACCGTTGG  (*EcoR*I) |  |
| MBP-PsRGL1-F | gagggaaggatttcacatatgATGGCCACCTACGACCCTG  (*Nde*I) |  |
| MBP-PsRGL1-R | ttaattacctgcagggaattcCAACACATTATGATTTATTATTCTACTTGCA  (*EcoR*I) |  |
| MBP-PsGAIP-B-F | gagggaaggatttcacatatgATGAAGAGAGACCACCATAGTCTTCA  (*Nde* I) |  |
| MBP-PsGAIP-B-R | ttaattacctgcagggaattcGTGGGTAGGTCTTGCTTTACAACTG  (*EcoR* I) |  |
