# Supplementary figures and images for: PsRGL1 negatively regulates chilling- and gibberellin-induced dormancy release by PsF-box1-mediated targeting for proteolytic degradation in tree peony
Source: Hortic Res. 2023 Mar 13;10(5):uhad044. doi: 10.1093/hr/uhad044 (PMC10541556; doi:10.1093/hr/uhad044)

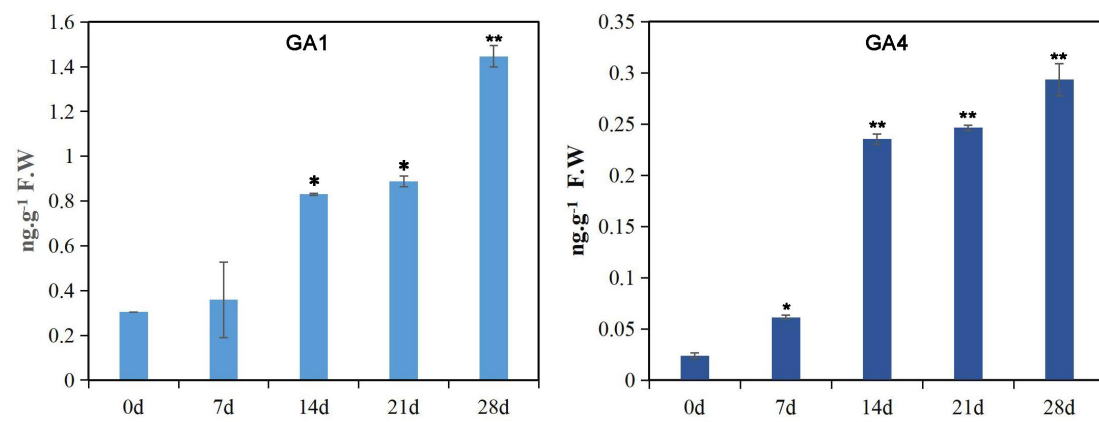

Fig. S1

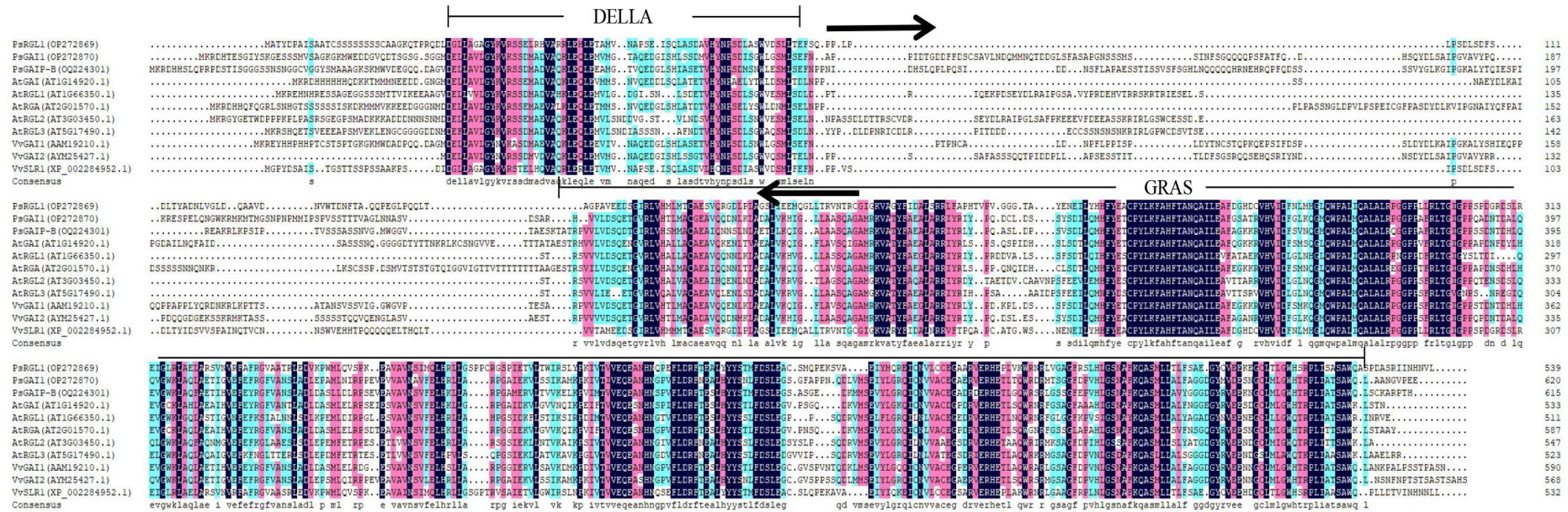

Fig. S2

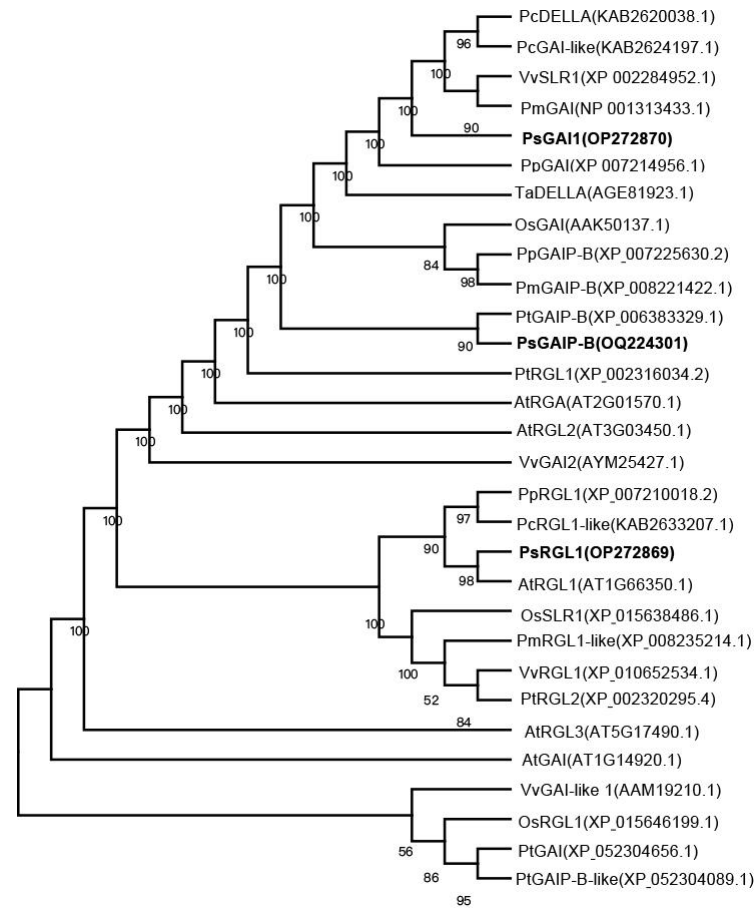

Fig. S3

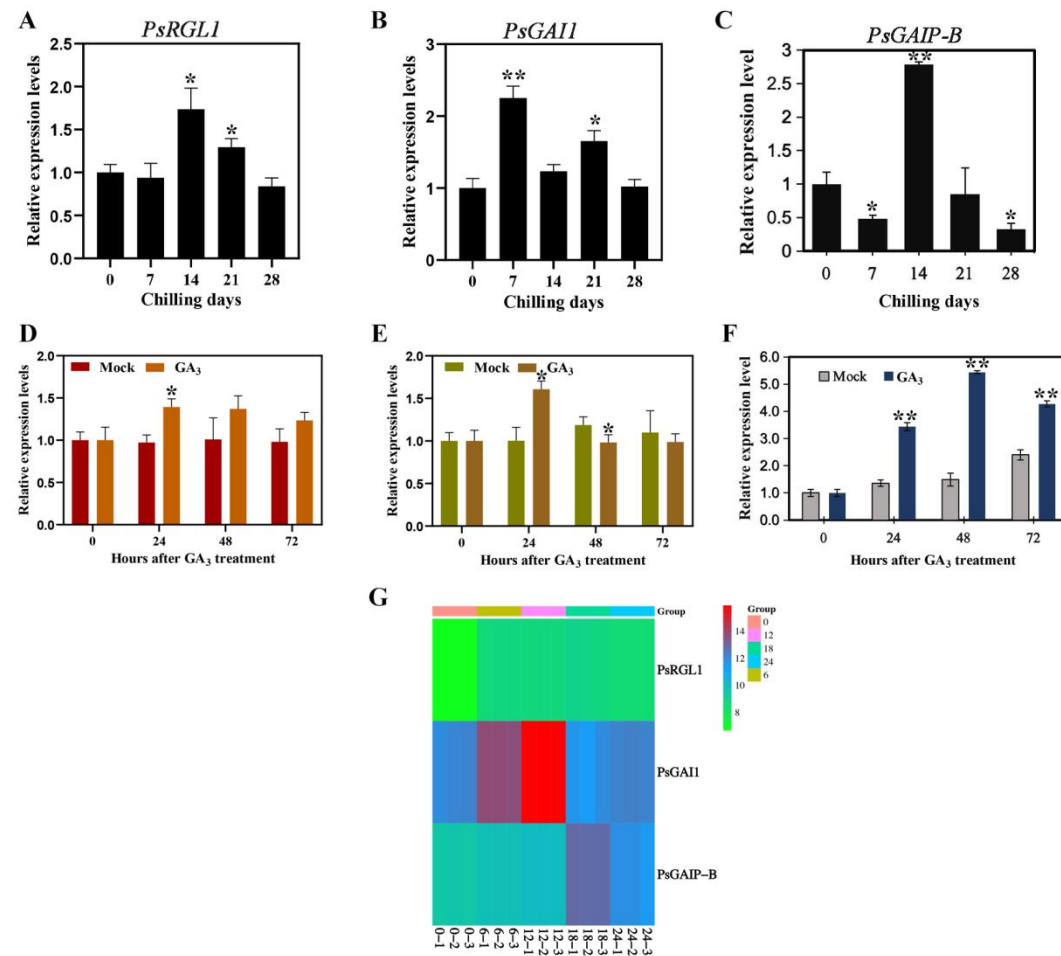

Fig. S4

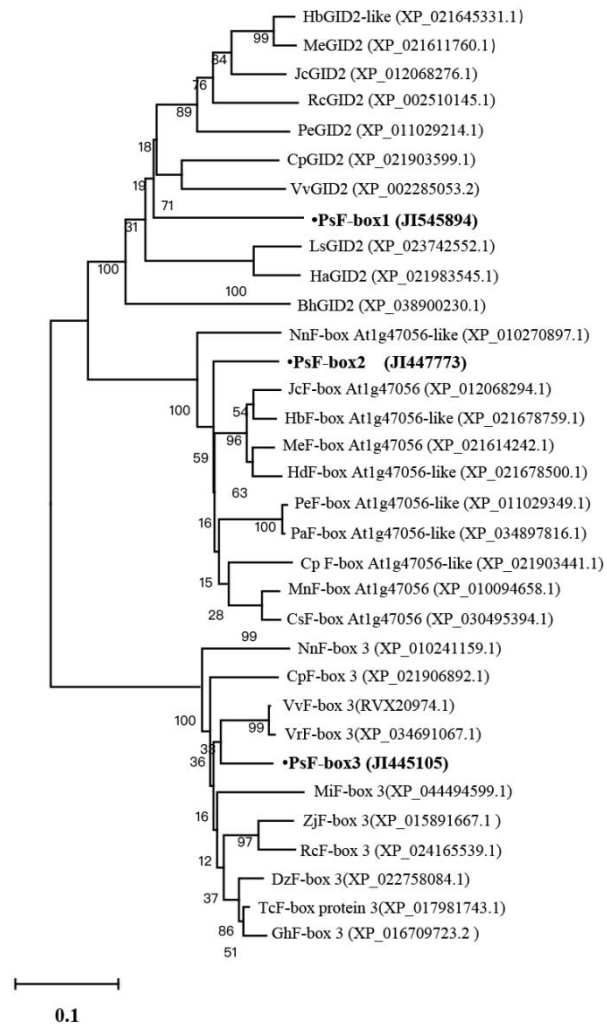

Fig. S5

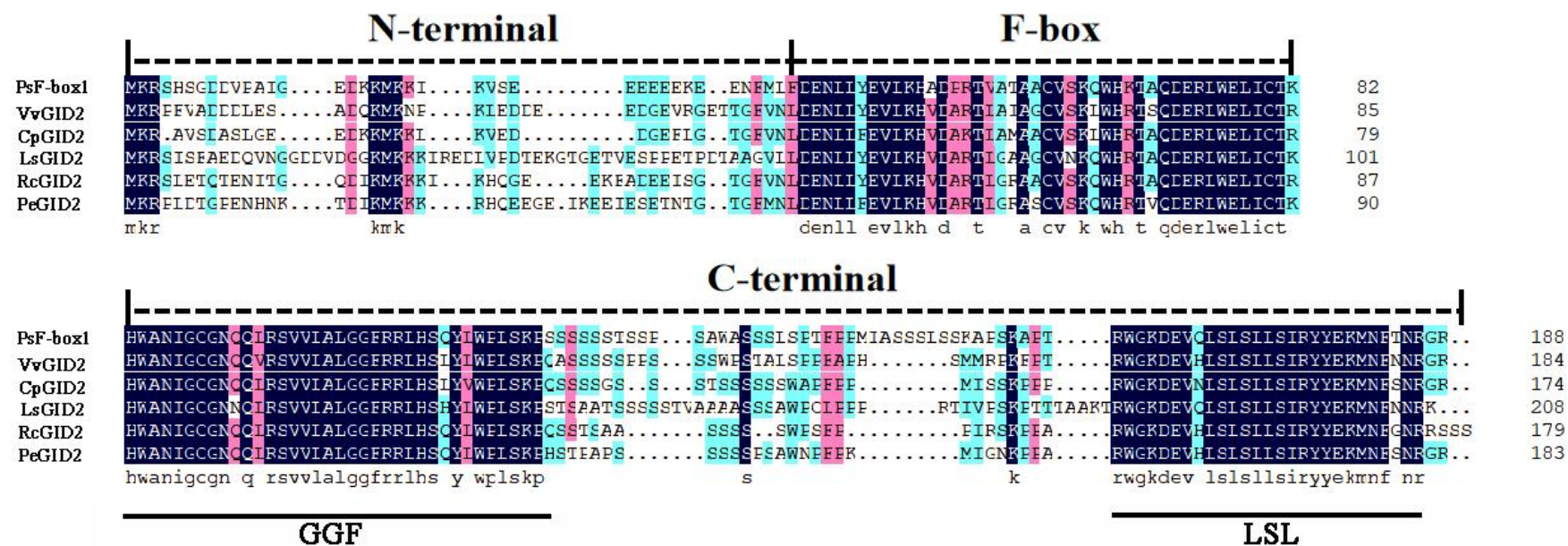

Fig. S6

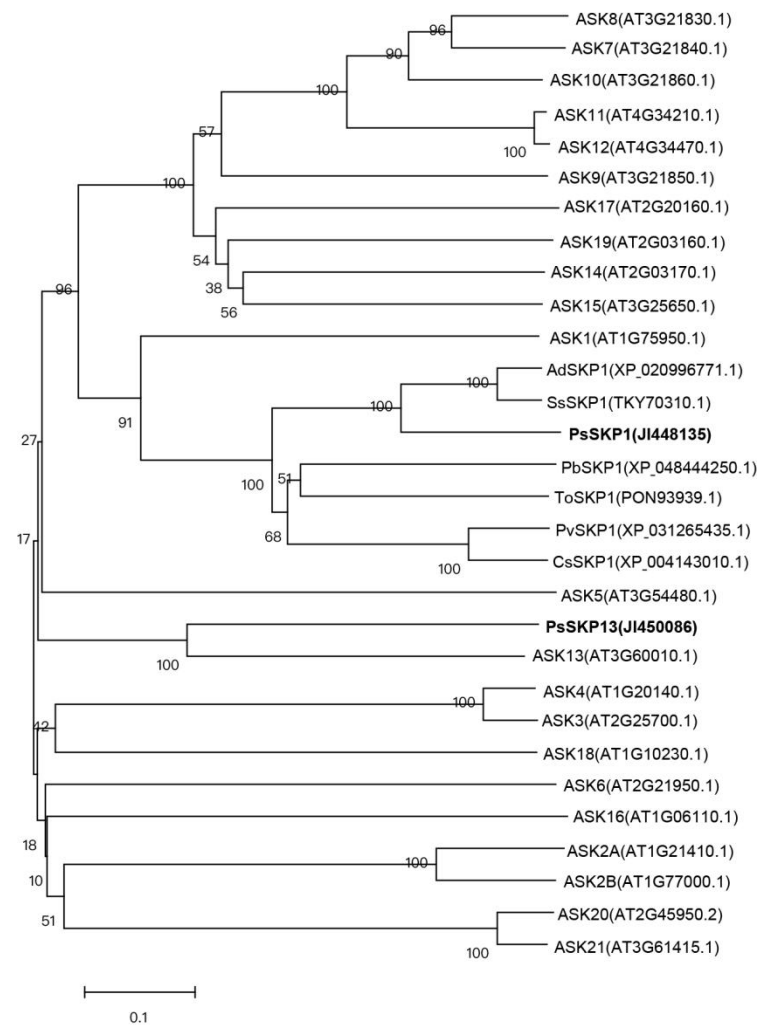

**Fig. S7**

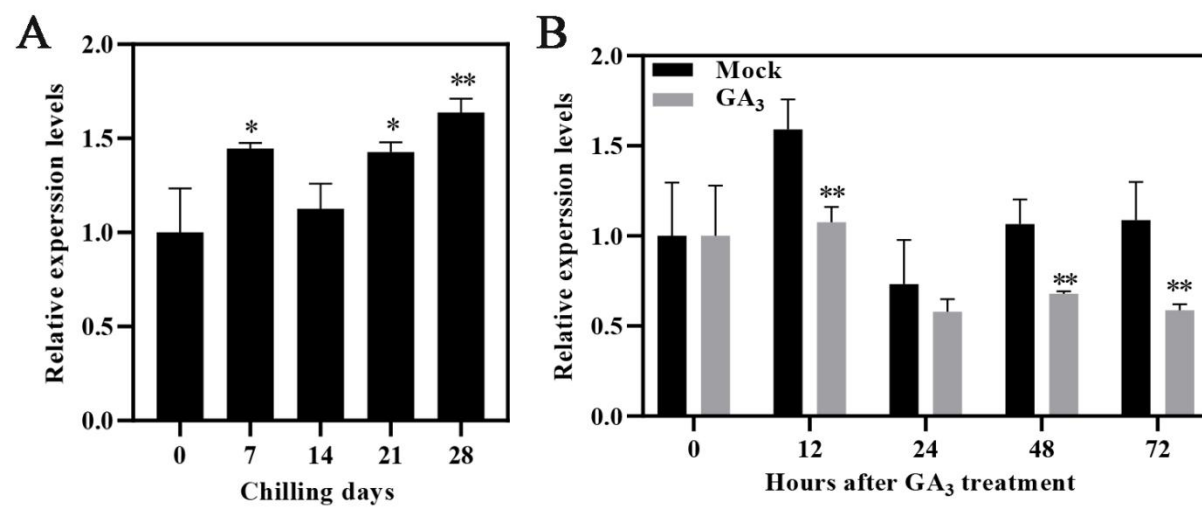

Fig. S8

Supplement: Web_Material_uhad044 [file web_material_uhad044.zip › Fig. S1-S8.pdf]
